# SnRNA-seq Interprets Mechanisms by which Three Glial Cell Types Influence Myelin Regeneration in Adult Drug-Resistant Epilepsy-Related Cognitive Impairment

**DOI:** 10.1007/s12035-025-05206-8

**Published:** 2025-07-17

**Authors:** Lu Wang, Jia-Qi Ma, Yong-Qian Bian, Xiao-Peng Qu, Yue Zhang, Chao Wang, Guo-Dong Gao, Long-Long Zheng, Qi-Xing Fang, Li-Jia Song, Liang-Liang Shen, Bei Liu

**Affiliations:** 1 Department of Neurosurgery, Tangdu Hospital, Airforce Military Medical University, Xi’an, Shaanxi China; 2 Department of Plastic and Burn Surgery, Tangdu Hospital, Airforce Military Medical University, Xi’an, China; 3 Department of Pediatrics, Tangdu Hospital, Airforce Military Medical University, Xi’an, China; 4 Department of Biochemistry and Molecular Biology, Basic Medical Science Academy, Airforce Military Medical University, Xi’an, China

**Keywords:** Drug-resistant, Epilepsy, SnRNA-seq, Cognitive impairment, Myelin sheath regeneration, Oligodendrocytes

## Abstract

Myelin regeneration has been shown in previous studies to ameliorate varying degrees of cognitive impairment in patients with neurodegenerative disorders such as epilepsy. The problem of myelin regeneration in adults with drug-resistant status epilepticus is a major key to the difficulty of treating cognitive impairment in adults with drug-resistant epilepsy (DRE). The purpose of this study is to provide a molecular map of myelin-related molecules under the cognitive deficits seen in DRE. We used a lamotrigine-pentylenetetrazol-resistant epilepsy mouse model and verified the cognitive problems and myelin changes using a water maze and conventional molecular biology techniques. We then analyzed the OLs in the hippocampus of the mice and the effect on myelin using sn RNA-seq technology. We found that the problem of cognitive impairment in drug-resistant epileptic mice is due to altered myelin plasticity. OL maturation induces pathological myelin regeneration which ultimately leads to cognitive impairment. The three glial cell types are closely related to the occurrence of myelin regeneration and jointly promote pathological myelin regeneration. Our study revealed the presence of myelin regeneration in DRE. All of this evidence suggests that normal myelin regeneration contributes to cognitive impairment improvement, but pathological myelin regeneration impairs cognition.

**Graphical Abstract:**

Schematic representation of myelin regeneration in cognitive impairment in adult drug-resistant epilepsy. Boxed are pathways or molecules related to the promotion of myelin regeneration in which a subpopulation is involved. Red font indicates promotion, blue font indicates inhibition, and gray font indicates unknown but relevant. 
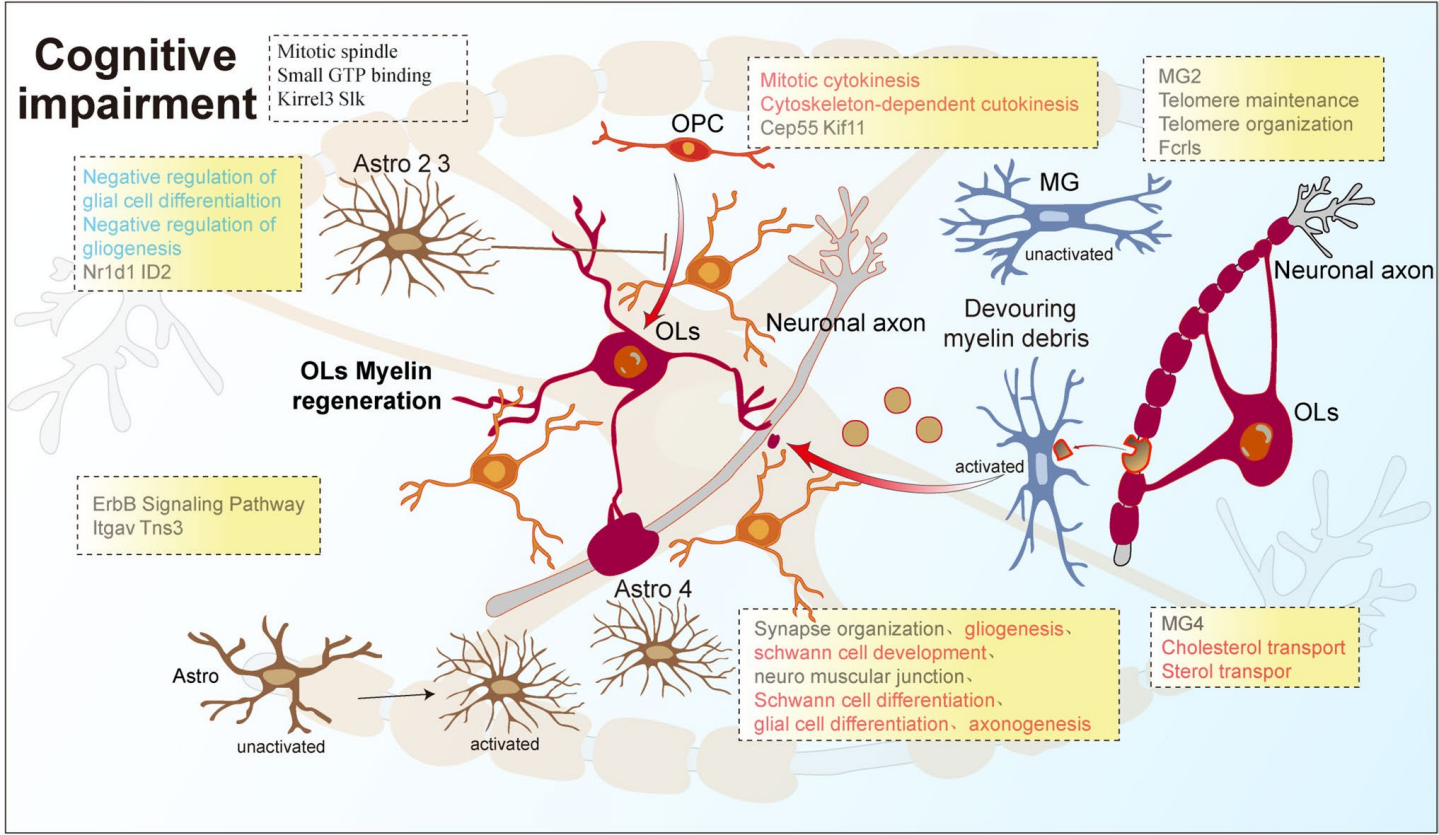

**Supplementary Information:**

The online version contains supplementary material available at 10.1007/s12035-025-05206-8.

## Introduction

Epilepsy is a chronic neurological disease that imposes a heavy economic burden on patients and their families [1]. Its pathological mechanism is neuronal apoptosis, neuroinflammatory response, neurotransmitter imbalance, and oxidative stress. Epilepsy significantly increases the risk of cognitive impairment [2] and seriously affects the quality of life of patients. Therefore, understanding the pathogenesis of cognitive impairment in epilepsy will greatly advance modern healthcare. Patients with DRE commonly have cognitive deficits, and myelin, which is closely related to oligodendrocytes (OLs), has long been recognized as critical for memory formation [3]. The microenvironment of the brain in status epilepticus can alter myelin plasticity by myelin cleavage, myelin layer detachment, myelin hyperplasia or thinning, and axon degeneration, among others, affecting the differentiation and proliferation of oligodendrocyte precursor cells (OPCs), inducing memory deficits [4, 5].

Epileptic cognitive impairment affects a large portion of the adult population [6]. Evidence suggests that adults have minimal nerve regeneration after CNS injury, and myelin regeneration is difficult [7]. Myelin plasticity in adults manifests itself in the form of subtle changes from the generation of entirely new myelin cells to changes in myelin sheath length, thickness, and distribution along axons [8]. Myelin sheaths are more often produced de novo by OPCs, with pre-existing OLs playing a lesser role, so the ability to produce de novo myelin sheaths after adult brain damage becomes even more important for the improvement of memory deficits [9]. Our recent studies have revealed that the ability to regenerate myelin sheaths also exists in the adult brain after damage and that there is neural repair [9]. Some of the other ways in which glial cells contribute to myelin sheath regeneration include the enhanced ability of OPCs to proliferate or differentiate; secretion of neurotrophic factor such as BDNF by astrocytes [10], which promotes the differentiation of OPCs thereby contributing to myelin regeneration [11]; and microglia (MG) promotes the removal of myelin debris and dead cells, cholesterol metabolism, and myelin regeneration by enhancing their own phagocytosis [12, 13]. Our finding that these pathways are also present in adult epileptic mice is not in agreement with previous theories on the difficulty of myelin regeneration in adult brain-damaged populations, but is similar to the most recent studies that appear to offer new therapeutic ideas for the amelioration of memory deficits in these populations [14]. However, exactly which molecules and pathways are used by the three glial cells in their effect on myelin regeneration in the context of DRE pathology is unknown. The development of SnRNA-seq technology has also prompted us to explore this intrinsic molecular change to help search for target molecules. Thus, focusing on target molecules involved in myelin regeneration may be key to finding improvements in adult cognitive impairment [15].

By analyzing differentially expressed molecules and pathways in the hippocampal OLs spectrum (OLs + OPCs) after DRE screened for networks involved in changes in myelin plasticity related to myelin plasticity, screening for cholesterol metabolism and (OPC) differentiation pathways; and then linking microglia and astrocytes to analyze the key molecules involved in regulating myelin regeneration in adults. This will help elucidate the possible regulatory networks involved in myelin shedding and regeneration, and thus, illustrate the bidirectional roles of the three glial cell subpopulations on myelin regeneration in adult DRE and key myelin regeneration targets.

## Experimental Section/Methods

### Specimen Collection from Epilepsy Patients

Our research is based on a previously published paper by our research group [16]. All specimens for this study were obtained from the Epilepsy Center of Neurosurgery, Tangdu Hospital, Fourth Military Medical University, which was approved by the Medical Ethics Committee of Tangdu Hospital (No. 202102–15) for the study protocol. Samples of surgical resection for epilepsy in patients with mild cognitive impairment (50–69 points) and moderate cognitive impairment (35–49 points) were screened based on the Wechsler Intelligence Scale scores of the patient's preoperative data, and all specimens were used with the informed consent of the patients or legally close relatives. We ended up with a randomized sample of 6 cases with epileptic cognitive impairment. Brain tissue processing and how controls were matched are cited in our previous publication [17].

### Preparation of DRE Mice

8-week-old male C57BL/6 J mice were purchased from the Laboratory Animal Center of the Fourth Military Medical University, with a body mass of (25 ± 5) g. The mice were housed in the neurosurgery laboratory of Tangdu Hospital and were kept in a 12-h light/dark alternation (8:00 am and 8:00 pm), and the mice were allowed to access to food and water freely, and were acclimatized for 1 week, and then in the ninth week, we started to carry out the preparation of the drug-resistant epilepsy model. We used the lamotrigine-pentylenetetrazole [18]. ignition method (lamotrigine 5 mg/kg; pentetrazol 40 mg/kg), injected every other day for a total of 35 days, and three consecutive times to achieve level IV and above was considered as successful ignition. A total of 70 mice were injected. After 1 day of new object recognition experiments and 5 days of water maze experiments taken after the end of the water maze experiments, 3 vs 3 SnRNA-seq experiments were done using mice that had found the platform a total of 0 times. Priority lamotrigine was injected, followed by pentylenetetrazole 30 min later.

### NOR Experiment

This experiment was taken from 70 drug-resistant epilepsy cognitive impairment (RE-CI) mice and 70 Cont mice for the new object recognition experiment. The mice were all 15 weeks old. A total of three objects were prepared, two identical and one different. At the beginning of the experiment, the mice were placed between the two objects, and the recorder was turned on to count the number of times, times, and distances that the mice explored the old and new objects, the number of times, times and distances that the mice moved around the old and new objects. The mice were thus tested for cognition, and if the mice had poor cognitive ability, there was no difference in the exploration of the old and new objects. If the mice had normal cognitive abilities, they explored the new object for a longer time than they explored the old object. The Cognitive Index (CI) is a measure of cognition. The Recognition index (RI) is calculated as RI = New Object/(New Object + Old Object) × 100%.

### Water Maze

A 15-week-old DRE mouse model of 70 mice with 70 Cont mice was used for water maze testing. We chose a quiet, light-proof, constant-temperature environment for the test. A bucket with a diameter of 120 cm and a depth of 1 m (including a round platform) was used. The experiment lasted for 5 days, with the first 4 days being the training period and the 5th day being the experimental period. The furthest quadrant of the mouse platform quadrant was released on day 5, and the time and number of shuttles in and at the platform quadrant were counted.

### Screening of RE-CI Mice

Based on the water maze experiment, the number of times crossing the platform was provisionally defined as 0 for cognitively impaired mice. Meanwhile, referring to the results of the new object recognition experiment, mice with low and statistically different cognitive index RI were screened to comprehensively identify the cognitively impaired animal model. A total of 23 cognitively impaired mice were screened.

### Myelin Regeneration Drug Therapy

Five 17-week-old RE-CI mice were selected for myelin regeneration treatment. Clemastine tablets were ground into powder form, then dissolved completely in DMSO and dissolved in drinking water, and administered to RE-CI mice at a concentration of 10 mg/kg/day for 60 days, and finally, the degree of recovery from cognitive impairment was detected by the water maze test.

### Transmission Electron Microscopy

Hippocampal tissues from two 17-week-old RE-CI and Cont mice each were selected for projection electron microscopy. After saline perfusion the tissue was preserved in electron microscopy fixative, embedded in Epon, and ultrathin sections were cut using an ultrathin sectioning machine and observed under a transmission electron microscope.

### Immunofluorescence Experiment

Mouse brains perfused with pre-cooled saline and 4% paraformaldehyde were fixed in 4% paraformaldehyde for 6–8 h, followed by dehydration with 30% sucrose and stored at 4 °C throughout. After the tissues had sunk sugar, 25um frozen sections were prepared and stored at −20℃. Sections were titrated with mouse anti-RB1CC1 (1: 250; proteintech, 17,250–1-AP), anti-Tns3 (1: 250; proteintech. 20,053–1-AP), anti-ID2 (1: 250; proteintech, Ag27134), anti-Becn1 (1: 250; proteintech, 11,306–1-AP), anti-Bbs9 (1: 250; proteintech, 14,460–1-AP) and other primary antibodies at 4 °C overnight. After rewarming for 2 h PBS was used to wash. Samples were then incubated with the secondary antibodies of Alexa Fluor 488/594 Donkey anti-Mouse/Rabbit IgG (1:400, Invitrogen) at room temperature for 4 h. Drops of DAPI were added to re-stain the nuclei and finally, the sections were allowed to air-dry and then sealed with the anti-quenching sealer, and stored at −20℃. Each molecule was stained with 2 mice each.

### Immunohistochemistry

Specimens were obtained from 6 each of posthumous donations (Cont) from patients with traumatic brain injury and surgical resection of cortical tissue from patients with cognitive impairment in drug-resistant epilepsy (RE-CI) in the specimen bank of Tangdu Hospital. Paraffin sections of 5 μm were prepared after dehydration. Subsequently, the slices were baked, dew-axed, closed, titrated with primary and secondary antibodies, color developed, and re-stained with nuclei. The final images were taken under a 20 × lens and magnified 6.6 ×.

### Single Nucleus RNA Sequencing (SnRNA-seq)

#### Hippocampal Tissue Sampling

Three 16-week-old RE-CI and Cont mice from each of the above screenings were taken and perfused with pre-cooled saline for cardiac perfusion, and the entire mouse brain was removed and stripped of the remaining tissues, with bilateral hippocampal tissues retained, blotted dry on filter paper, rapidly frozen in liquid nitrogen, and subsequently stored at −80 °C, with the entire process performed on ice. This part of the tissue will be used to perform SnRNA-seq experiments.

### Nuclei Isolation Sorting from Hippocampal Tissues

Hippocampal tissues were harvested from drug-resistant epilepsy with cognitive impairment and normal mice and were washed in pre-cooled PBSE (PBS buffer containing 2 mM EGTA). Nuclei isolation was carried out using GEXSCOPE® Nucleus Separation Solution (Singleron Biotechnologies, Nanjing, China) refer to the manufacturer's product manual. Isolated nuclei were resuspended in PBSE to 10^6^ nuclei per 400 μl, fltered through a 40 μm cell strainer, and counted with Trypan blue. Nuclei enriched in PBSE were stained with DAPI (1:1,000) (TermoFisher Scientifc, D1306). Nuclei were defined as DAPI-positive singlets.

### Single Nucleus RNA-Sequencing Library Preparation

The concentration of singlenucleus suspension was adjusted to 3–4 × 10^5^ nuclei/mL in PBS. Single nucleus suspension was then loaded onto a microfluidic chip (GEXSCOPE® Single NucleusRNA-seq Kit, Singleron Biotechnologies) and snRNA-seq libraries were constructed according to the manufacturer’s instructions (Singleron Biotechnologies). The resulting snRNA-seq libraries were sequenced on an Illumina novaseq 6000 instrument with 150 bp paired end reads.

### Primary Analysis of Raw Read Data (SnRNA-seq)

Process the original reads using CeleScopev1.5.2 (Samsung Biotechnology) with default parameters to generate a gene expression profile. Simply put, extract barcodes and UMIs from R1 reads and perform calibration. Trim the adapter sequence and poly A tail from R2 reads, and compare the trimmed R2 reads with the grcm38 (mm10) transcriptome using STAR (v2.6.1b). Then assign the uniquely located reads to genes with feature counts (v2.0.1). Group successfully assigned Reads with the same cell barcode, UMI, and gene, and generate a gene expression matrix for further analysis.

### Quality Control, Dimension-Reduction, and Clustering (Scanpy)

Use Scanpy v1.8.1 for quality control, dimensionality reduction, and clustering in Python 3.7. For each sample dataset, we filter the expression matrix according to the following criteria: 1) exclude cells with gene counts less than 200 or the top 2% of gene counts; 2) Excluding cells in the top 2% of UMI count; 3) Excluding cells with mitochondrial content > 10%; 4) Exclude genes that express less than 5 cells. After filtration, 61,215 cells were retained for downstream analysis, with an average of 1293.07 genes and 2468.09 UMIs per cell. Normalize the original count matrix based on the total count of each cell and logarithmically convert it to a normalized data matrix. By setting the flavor to'flavor', the top 2000 variable genes were selected. Perform principal component analysis (PCA) on the variable gene matrix for proportional analysis, using the top 20 principal components for clustering and dimensionality reduction. Using the Leuven algorithm, set the resolution parameter to 1.5 and divide the cells into 23 clusters. Visualize cell clusters by using Unified Manifold Approximation and Projection (UMAP) {t-Distribution Random Neighborhood Embedding (t-SNE)} [16].

### Batch Effect Removal

Harmony: Use Harmony v1.0 to remove batch effects between samples using the first 20 principal components in PCA.

### Differentially Expressed Genes (DEGs) Analysis (scanpy)

To identify differentially expressed genes (degrees), we used scanpy.tl.rank_ Genes_ The groups() function is based on the Wilcoxon rank sum test and default parameters, and selects cell groups with gene expression exceeding 10% and an average log (fold change) value greater than 0.25 degrees. The adjusted *p*-value was calculated using Benjamini Hochberg correction, with 0.05 as the standard for evaluating statistical significance.

### Pathway Enrichment Analysis

To investigate the potential functions of DEGs, we used Gene Ontology (GO) and Kyoto Encyclopedia of Genes and Genomes (KEGG) analysis, in conjunction with the"Cluster Analyzer"R package v3.16.1. P_ Pathways with an adj value less than 0.05 are considered significantly enriched. The selected significance path is plotted as a bar chart. In the GSVA pathway enrichment analysis, the average gene expression of each cell type was used as input data (reference). Using the gene ontology gene set, including molecular function (MF), biological process (BP), and cellular component (CC) categories as references.

### Cell-Type Recognition with Cell-ID

Cell-ID is a multivariate method that extracts the gene signature of each cell and uses hypergeometric testing (HGT) for cell identity recognition. By using multiple correspondence analysis to reduce the dimensionality of the normalized gene expression matrix, both cells and genes are projected onto the same low dimensional space. Then, calculate the gene sequencing of each cell to obtain the most characteristic gene set for that cell. SGT is an HGT analysis of the brain reference gene set in the SynEcoSys database, which contains characteristic genes for all cell types. The identity of each cell is determined to have the minimum HGT p-value for the cell type. For cluster annotations, calculate the frequency of each cell type in each cluster and select the cell type with the highest frequency as the cluster identifier.

The cell type identification of each cluster is determined based on the expression of typical markers in the reference database SynEcoSysTM (Singleton Biotechnology). SynEcoSysTM contains a collection of typical cell type markers for single cell sequence data, sourced from CellMakerDB, PanglaoDB, and recently published literature. Typical markers and their corresponding cell types are listed in TableS3.

### Subtyping of Major Cell Types

In order to extract Astrocytes/OLs/OPCs/Microglia from specific clusters, a high-resolution map of cells was obtained, and the clustering resolution was set to 0.5/1/0.3/0.6 for further detailed analysis.

### Filtering Cell Doublets

Estimate cell dimorphism based on the expression patterns of typical cell markers. Any cluster rich in multiple cell type-specific markers is excluded for downstream analysis.

### Pseudo Time Trajectory Analysis: Monocle2

The cell differentiation trajectory of monocyte subtypes was reconstructed using Monocle2 v 2.10.0 (reference). When constructing trajectories, the Seurat (v3.1.2) feature () is used to select the top 2000 highly mutated genes, and DDRTree() is used for dimensionality reduction. Through plot in monocle, 2_ Cell_ The trajectory () function visualizes the trajectory.

### Cell Cycle Analysis

Calculate the cell cycle score for each cell using the cell cycle score function implemented in the Seurat v 3.1.2 software package.

### Cell–Cell Interaction Analysis (CellChat)

Analyze the intercellular communication network from SnRNA-seq data using CellChat (version 0.0.2). A CellChat object was created using the R package process. The cell information is added to the meta slot of the object. Establish a database of ligand-receptor interactions and perform matching receptor inference calculations.

### ScMetabolism

Metabolism (v0.2.1), an R-package designed for quantitative analysis of single-cell metabolic activity. Collect KEGG metabolic pathways and visually calculate metabolic pathway enrichment scores. Visualize the rating of a specific pathway through the feature map ()/VlnPlot() functions in Seurat and feature map ().

### Statistical Analysis

The comparison of cell distribution between the two groups was conducted using an unpaired double-tailed Student *t*-test. Compare two groups of cells using unpaired gene expression or gene signatures for double tailed Wilcoxon rank sum test. All statistical analysis and statements were conducted using r. The statistical test used in the Figure is represented by a legend, with a statistical significance of *p* < 0.05. The exact value of n is shown in the figure and legend, and the representation of n is shown in the legend.

Except SnRNA-seq-related statistical methods (a detailed description exists in Material Methods), unpaired two-sided *t*-tests were used to analyze immunofluorescence, immunohistochemistry, Western Blot, and water maze results, and the data were analyzed for graphing using GraphPad Prism 8 and ImageJ. A value of *P* < 0.05 was considered statistically significant (designated by a single asterisk; double asterisk, *P* ≤ 0.01; triple asterisk, *P* ≤ 0.001).

### Data Availability

Raw sequence and processed data of single-nucleus RNA sequencing for this study are available on the Gene Expression Omnibus database (GEO) under the identifier GSE241349. Raw image files used in the Figures that support the findings of this study are available from the corresponding authors upon reasonable request.

### Supporting Information

Supporting Information is available from the Wiley Online Library or the author.

## Results

### OLs and Myelin Regeneration on Cognitive Impairment in Adult DRE

**Fig. 1:**
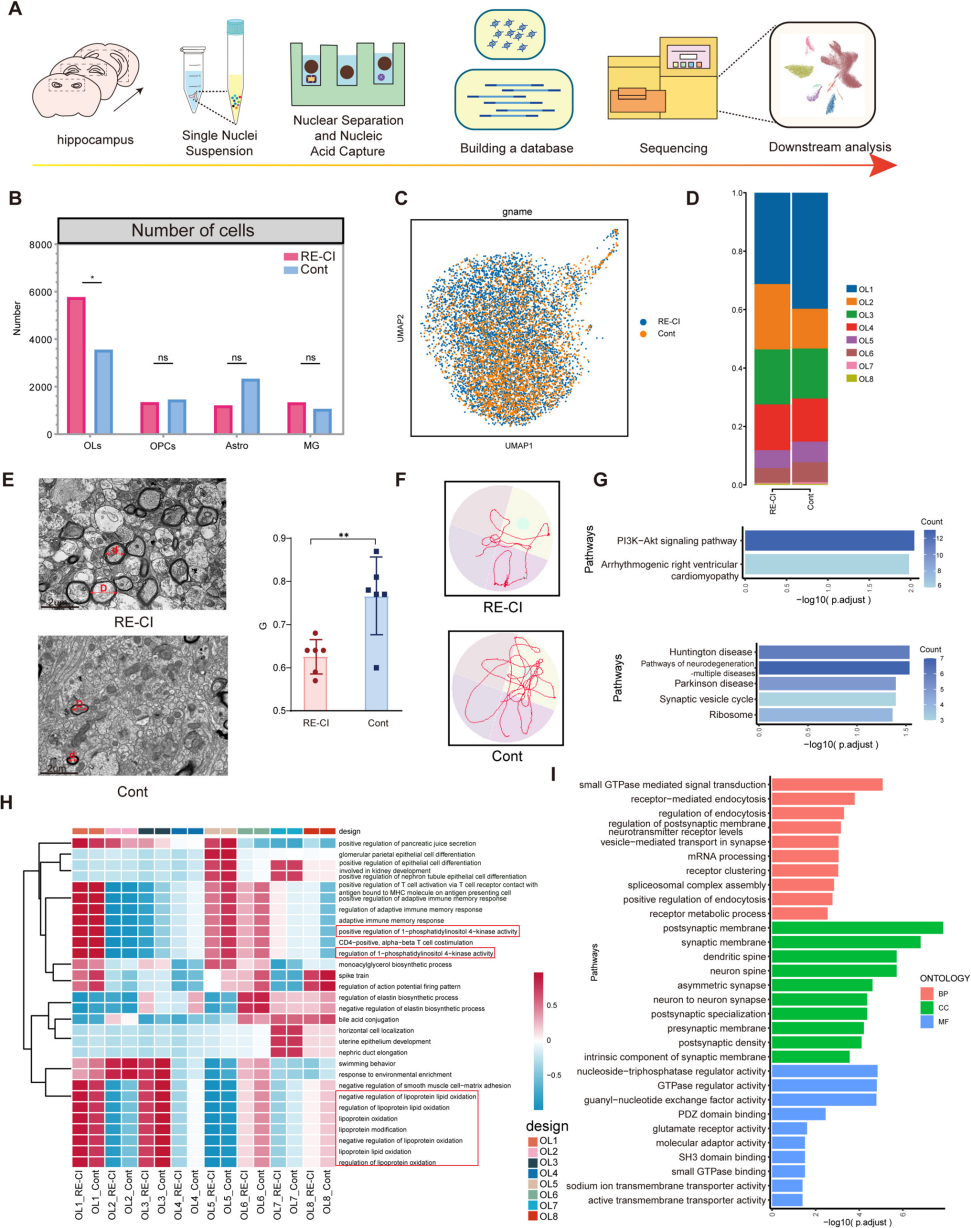
Oligodendrocytes and myelin regeneration in cognitive impairment in adult drug-resistant epilepsy. **A**) Single nucleus RNA sequencing process: take the bilateral hippocampal tissues of RE-CI and Cont mice to extract and isolate cell nuclei to establish DNA libraries, and carry out single nucleus sequencing to differentiate cell subgroups by downward dimensional clustering. **B**) Intergroup analysis histograms of cell counts of the glial cells (OLs, OPCs, Astrocytes, and Microglia). Using bilateral non-paired *t*-test. **C**) Uniform flow shape approximation and projection (UMAP) plot of oligodendrocytes between groups. **D**) Histogram of the analysis of the percentage of cell numbers between subgroups OL1-8, with different colors representing different subgroups. **E**) Electron microscopy analysis of hippocampal region of brain tissue in RE-CI vs Cont mice. (d: inner diameter of myelin-coated axons. **D**: outer diameter of myelin-coated axons). Statistical plots show the values of d/D between groups, values are shown as mean ± standard deviation, two-sided *t*-test was used, **P < 0.01. **F**) Representative water maze behavioral test of RE-CI and Cont mice, RE-CI mice have cognitive impairment. **G**) Te upper graph represents the result of KEGG term enriched by OLs upregulation, and the lower graph represents the result of KEGG term downregulated by OL1 the horizontal coordinates are—log10(p.adjust), vertical coordinate is GO Term, the longer bar represents the more significant enrichment result. **H**) Showing the GSVA scoring results of intergroup pathways of OL1-8 subgroups, the vertical axis is the name of each pathway, and the horizontal axis is the subgroups of OL1-8. **I**) Enrichment analysis of GO upregulated pathways for OLs overall, including BP, CC, and MF

We used bilateral hippocampal tissues from adult drug-resistant epileptic memory-impaired mice versus normal mice for snRNA-seq analysis (Fig. 1A and Table S5) and found increased numbers of OLs and Microglia, decreased numbers of OPCs and astrocytes, and changes in their cell counts suggestive of possible hippocampal neuronal cytopathies (Fig. 1B–C and TableS1). Increased numbers of OLs appeared to be a response to the stress of cognitive impairment after DRE. The protective effect and the number of OLs strongly correlated with myelin regeneration capacity [19]. Altered myelin plasticity was also found in DRE with cognitive impairment mice. We counted the ratio G of the outer diameter of myelinated axons (including myelin) to the inner diameter of axons to calculate the degree of myelin change, and the results showed that the ratio G decreased in drug-resistant epileptic memory-impaired mice, suggesting an increase in the thickness of myelin sheaths, with a significant difference that allowed myelin plasticity to be altered (Fig. 1E and TableS2). To specifically dissect the contribution of OLs to myelin plasticity in adult memory disorders, they were subdivided into eight subpopulations of varying proportions, and we found that the number of OL 1 and 2 were more significantly changed, which may have an important role in myelin regeneration (Fig. 1D, and TableS3). OLs overall KEGG enrichment showed the upregulation of the PI3K-Akt signaling pathway, which involves related molecules such as the laminin Lama2, which plays a role in the coordination of neurotoxicity and neuroprotection, thus suggesting that there is an overall neuroprotective effect of OLs in drug-resistant epileptic memory-impaired mice (Fig. 1G, I) [20]. Pathway enrichment revealed the presence of a number of neuroprotective processes. These processes include the following: negative regulation of lipoprotein oxidation, lipoprotein oxidation, lipoprotein modification, negative regulation of lipoprotein antioxidation and lipoprotein oxidation, regulation of lipoprotein oxidation, positive regulation of 1-phosphatidylinositol 4-kinase activity and regulation of 1-phosphatidylinositol 4-kinase activity. These pathways are directly or indirectly involved in lipid metabolism, which is associated with myelin regeneration. Among them, the OL1 subpopulation was enhanced in the RE-CI group, suggesting that the OL1 subpopulation may play a neuroprotective function in promoting myelin synthesis in drug-resistant epileptic memory disorders, whereas the roles of OL3, 5, 6, and 8 were not significant in the groups (Fig. 1H). The role of OL1 in the KEGG-enriched processes showed a relevant neurodegenerative effect and showed downregulation of pathways related to neurodegenerative diseases and the presence of synapse-related GO terms, suggesting a protective role for OL1 (Fig. 1G, I).

Thus, the data tentatively suggest that increased OLs and myelin regeneration are present in adult epileptic memory disorders.

### OL Subpopulation Functionally Specific Molecules for Myelin Regeneration

**Fig. 2:**
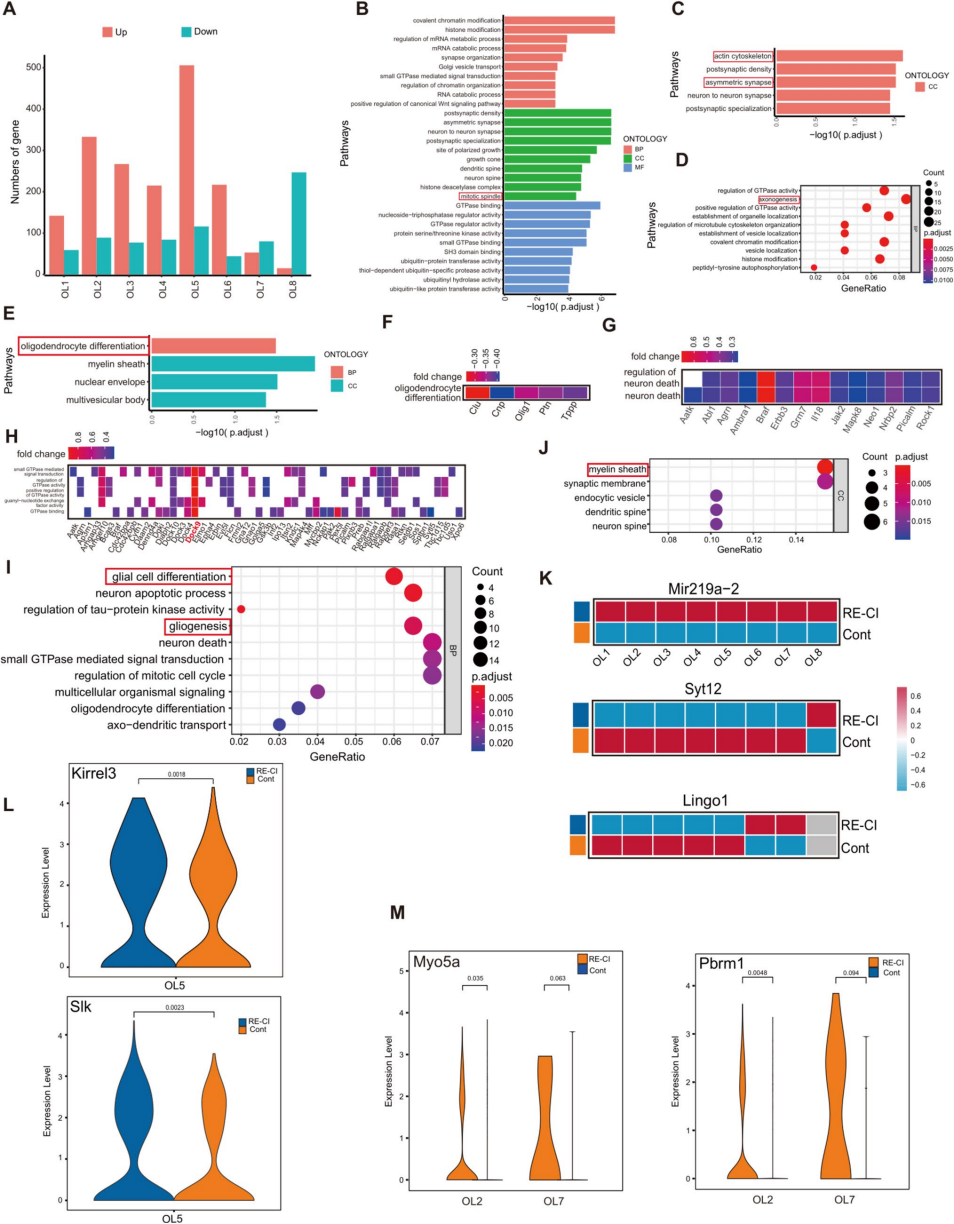
Oligodendrocyte subpopulation function-specific molecules promote myelin regeneration. **A**) Histogram of the results of inter-subgroup DEG difference analysis for OLs subgroups, the horizontal axis represents the 8 subgroups, the vertical axis represents the number of DEGs, red represents upregulation, and cyan represents downregulation. **B**) Upregulation GO enrichment analysis for OL5 subgroups showing the three parts of BP, CC, and MF, with the longer columns representing the more significant differences, and the vertical axis is GO Term. **C**) Upregulated GO enrichment analysis of OL7 subgroups, with longer bars representing more significant results. **D**) Bubble plot of upregulated GO enrichment analysis for the OL2 subpopulation. **E**) OL4 subpopulation downregulation GO enrichment analysis, longer bars represent more significant results. **F**) OL4 subpopulation downregulation GO network shows Olig1 is significantly involved in signaling pathways related to oligodendrocyte differentiation. **G**) OL2 subpopulation upregulation GO network shows GRM7 is significantly involved in signaling pathways regulating neuronal death. **H**) OL5 subpopulation upregulation GO network shows DOCK9 is significantly involved in in vivo GTP-related signaling pathway. **I**) Bubble plot of enrichment analysis of KEGG pathway upregulated by OL6 subgroup, circle size indicates the number of genes enriched to, and color represents *P*-value. **J**) Enrichment analysis of GO CC downregulated by OL6 subgroup, circle size indicates the number of genes enriched to, and color represents *P*-value. **K**) Intergroup in situ expression of Mir219a-2, Syt12 and Lingo1 molecules. **L**) Violin plots showing differential expression levels of Kirrel3 and Slk between groups. **M**) Violin plots showing significant differences in expression levels of Pbrm1 and Myo5a between subgroups OL1-8

OLs are the cells of myelin. We found a total of eight major cellular subpopulations after we processed OLs for dimensionality reduction clustering, of which the largest number change was in the OL1 and 2 subpopulations (Figure S1 and TableS3-S5). The number of cells does not necessarily mean functional differences, and differentially expressed gene (DEG) analysis can more accurately reveal gene expression differences related to function at the molecular level (Fig. 2A). We found that there are processes affecting the role of OL proliferation and differentiation, axonogenesis, autophagy, and myelin regeneration. Mir219a-2 is a non-coding RNA, which affects the post-translational modification of proteins, and its significant elevation in the RE-CI group suggests changes in gene expression of the subpopulation (Fig. 2K).

Lingo1 is an important negative regulator of OL differentiation and axonal myelin formation. Based on Lingo1 we performed a preliminary screen of eight major cell subpopulations and found that OL6 and 7 inhibited myelin regeneration. OL8, although not differentially but with its high expression of Syt11, promoted synaptic connectivity at tessellated fibers, which has been associated with memory transmission(Fig. 2K) [21]. OL6 and 7 inhibition of myelin regeneration is more prominent, as shown by the enrichment of the actin cytoskeleton, asymmetric synapses, and myelin sheath GO enrichment (Fig. 2C, I). OL6 exhibited a failure of myelin regeneration despite the presence of proliferation and differentiation (Fig. 2J). The OL4 subpopulation was directly targeted to inhibit the Olig1 and OL4 subpopulations, and inhibit OL1 expression, which is involved in the inhibition of the OL differentiation process, inhibition of OL development, and the myelin regeneration process (Fig. 2E, F) [22]. OL2 was significantly enriched to GO terms such as axonogenesis, regulation of neuron death, neuron death, neuronal pathology, and axonal degeneration. Highly enriched Grm7 directly impaired axon growth, leading to neuron death (Fig. 2D, G).

The above biological behavior of oligodendrocytes (OLs) caused by epileptic memory impairment is reliable. Although myelin regeneration occurs less frequently in adults. However, we observed the phenomenon of myelin regeneration in adult drug-resistant epilepsy memory impairment. The number of upregulated genes is most prominent in the OL5 subgroup, which prompted us to conduct in-depth analysis of their potential functions (Fig. 2A). The enrichment results showed that it was involved in the mitotic spindle process, where mitosis promotes the proliferation of the OL5 subpopulation, and the number of OLs represents the speed of myelin regeneration (Fig. 2B). This shows that the OL5 subpopulation promotes the myelin regeneration process. We would like to continue our search for target molecules with the most pronounced difference, Dock9, enriched to Small GTP binding terms (Fig. 2H). Dock9 as a contributor of GTP in spatial biological activity is important for maintaining the growth of neuronal synapses. OLs prolong the protrusion close to the axon forming an insulating membrane, which is wrapped around the outside of the axon to promote the propagation of axonal electrical signals [23]. In the OL5 subpopulation, the gene expression levels of Kirrel3 and Slk were significantly increased (Fig. 2L). Mossy fibers are important hubs connecting the dentate gyrus to hippocampal CA3, and determining the efficiency of memory transmission [24]. Kirrel3 is necessary for the production of the MF filopodial feet and is a molecule that plays an important role in the maintenance of the integrity of the neural circuits [25]. The high expression of Kirrel3 in OL5 maintains the integrity of the circuits. Myelin formation immediately follows and loop integrity alone is not sufficient to support memory regeneration. Myelin regeneration is significantly elevated in OL5, and Slk is a complex kinase that plays a role in OL differentiation and myelin production. Slk inhibitors lead to a decrease in myelin proteins such as MBPP, PO, and MAG [26]. High expression of Slk is one of the reasons why OL5 promotes myelin regeneration and OL5 is a subcluster of the adult subpopulation of memory maintenance in epilepsy.

The above has shown the presence of OLs and myelin regeneration in adult DRE memory deficits, but we found that these memory deficits did not improve after epilepsy. In other words, this myelin regeneration did not have a sizable protective effect. Therefore, we continued to analyze the differential molecules and found that in OL3, 5, and 7 subpopulations, Pbrm1 was expressed at significantly elevated levels in the RE-CI group, which is a negative regulator of cell proliferation and inhibits the proliferation of OL5 and 7 subpopulations. Myo5a was significantly enriched in OL2 and 7 subpopulations, which affects development and is present in OPCs and OLs. Myo5a is also associated with myelin peripheral axons, and may affect the function of OL myelin-related axons (Fig. 2M). We speculate that such myelin regeneration may not be protective and that there are changes in molecular expression that affect myelin function and remain non-functional.

### OPC Differentiation Capacity Is a Major Factor Influencing Myelin Regeneration

**Fig. 3:**
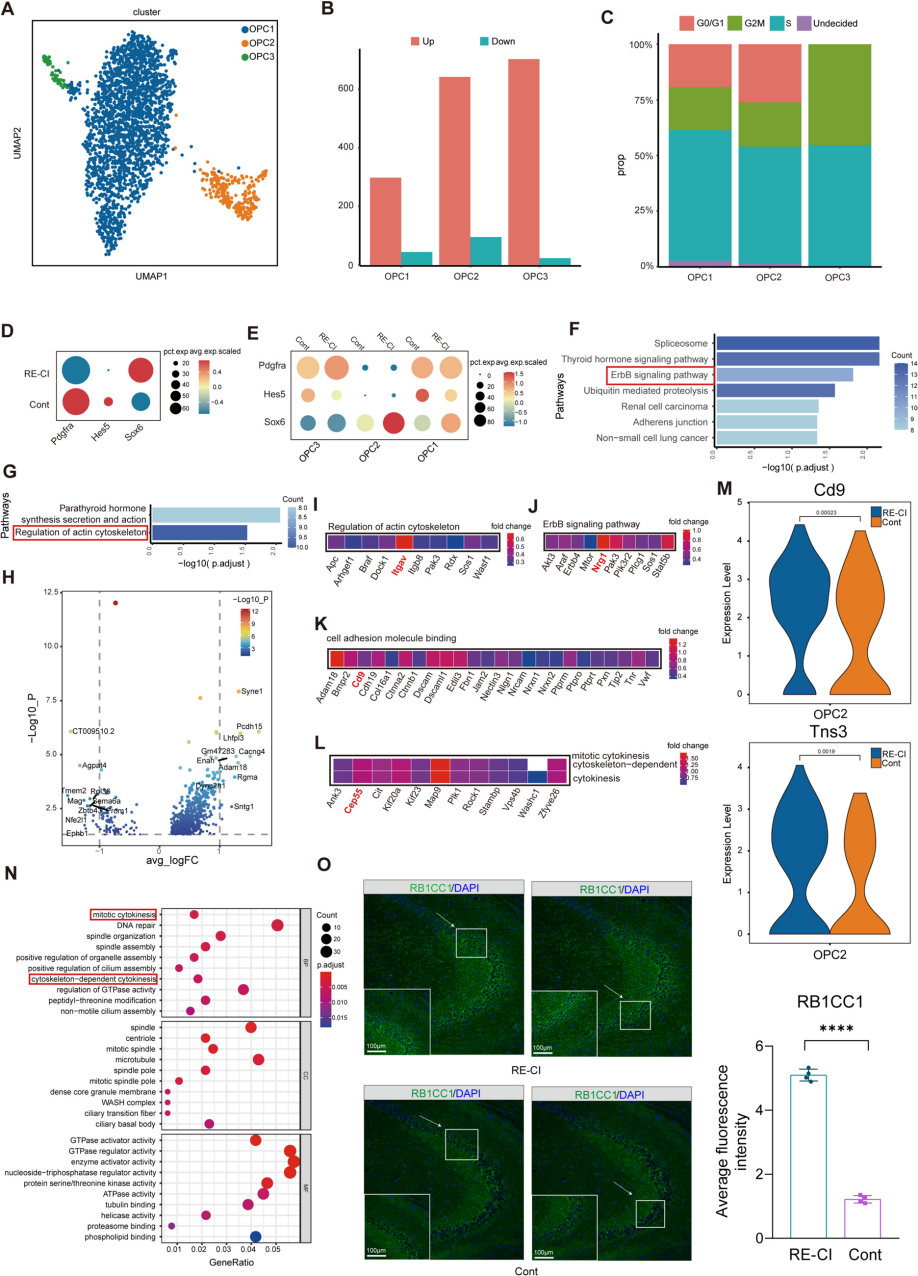
OPC differentiation capacity is a major factor influencing myelin regeneration. **A**) OPCs were subjected to Uniform Mobility Approximation and Projection (UMAP) analysis and divided into 3 subgroups. **B**) Histogram of the results of intergroup DEG differential analysis of OPC subgroups. **C**) Histogram of the percentage of cells in the S-phase, G2/M-phase, and G0/G1-phase for each cell type of OPC1-3, respectively. **D**) Intergroup OPC bubble plots of intergroup differential expression analysis of Pdgfra, Hes5, and Sox6. **E**) Bubble plots of intergroup differential expression analysis of Pdgfra, Hes5, and Sox6 in OPC1-3 subpopulations. **F**–**G**) Intergroup upregulated KEGG enrichment analysis, with the longer bar representing the more significant result (G-OPC1, F-OPC2). **H**) Volcano plot of inter-subgroup differential analysis of OPC2. **I**–**L**) Intersubpopulation intergroup upregulation of the GO network shows that key molecules are significantly involved in the signaling pathway (key molecules are labeled). **M**) Violin plot showing the differential expression levels between the Cd9 and Tna3 groups. **N**) OPC3 subgroup upregulation GO enrichment analysis showing BP, CC, and MF components, the longer bar represents the more significant difference, and the vertical axis is the GO Term. **O**) Analysis of immunofluorescence staining results of hippocampal frozen sections for RB1CC1 and MBP between groups, and the arrowheads show fluorescence signals at hippocampal sites

OPCs can differentiate into OLs, and the ability to promote regenerative differentiation of OPCs is closely related to myelin regeneration in OLs [27]. We, therefore, focused on OPC differentiation in adult epileptic memory disorders to search for the molecular basis of pro-myelin regeneration in adulthood. Based on dimensionality reduction clustering we categorized OPCs into three subgroups OPC1, 2, and 3 (Fig. 3A and TableS4). OPC1 was found to be the subpopulation with the largest percentage, followed by OPC2 and the least was OPC3 (Figure S2). The number of DEGs showed that OPC3 was the subpopulation with the highest number of upregulated gene, suggesting an important function of OPC3 (Fig. 3B). Next, we applied OPC cell cycle analysis to select the subpopulation with the strongest differentiation capability, and the results showed that OPC3 seemed to have all of its cells at the differentiation stage (Fig. 3C and TableS5). Pdgfra and Hes5 are homeostatic genes in OPCs, so we thought that OPC1 and 2 might be the subpopulation of homeostatic cells with normal proliferation and differentiation. OPC3 represented the subpopulation that receives the protective effect of demyelination stress (Fig. 3D).

Considering that Sox6 binds to the promoter of MBP to inhibit myelin production, the overall change in OPCs was shown to inhibit myelin regeneration (Fig. 3E). OPC2 promotes myelin regeneration in drug-resistant epilepsy with cognitive impairment, as evidenced by significantly increased expression of Tns3 and CD9 molecules. Tns3 can target Olig2 to promote the differentiation process of oligodendrocytes [27]. CD9 is involved in cell proliferation, differentiation, and regulation, and has the potential to promote myelin regeneration. CD9 can participate in maintaining the entire myelin sheath structure and promoting myelin sheath protection [28, 29] (Fig. 3I, K).

The presence of myelin disruption was expected, but enrichment of the ErbB signaling pathway of OPC2 and the integrin molecule ITGAV, which plays an important role, was found to promote cellular differentiation (Fig. 3H, G). OPC3 is enriched to mitotic cytokinesis and cytoskeleton-dependent cytokinesis, which are involved in promoting OPC division and proliferation, and thus, promote their differentiation (Fig. 3M). Among the pathways cross-linking molecular networks, we found that Cep55 and Kif11 play important roles (Fig. 3N). Cep55 promotes neural progenitor cell division [30] and overexpression of Kif11 would be beneficial in rescuing LTP in Alzheimer's disease, thereby restoring learning memory deficits [31]. Overexpression of both molecules seems to play a protective role in adult epileptic memory deficits that improve memory, and therefore, may serve as a molecular basis for targeted therapies.

### Differentiation and Transcriptional Regulation of OPCs in Cognitive Disorders

**Fig. 4:**
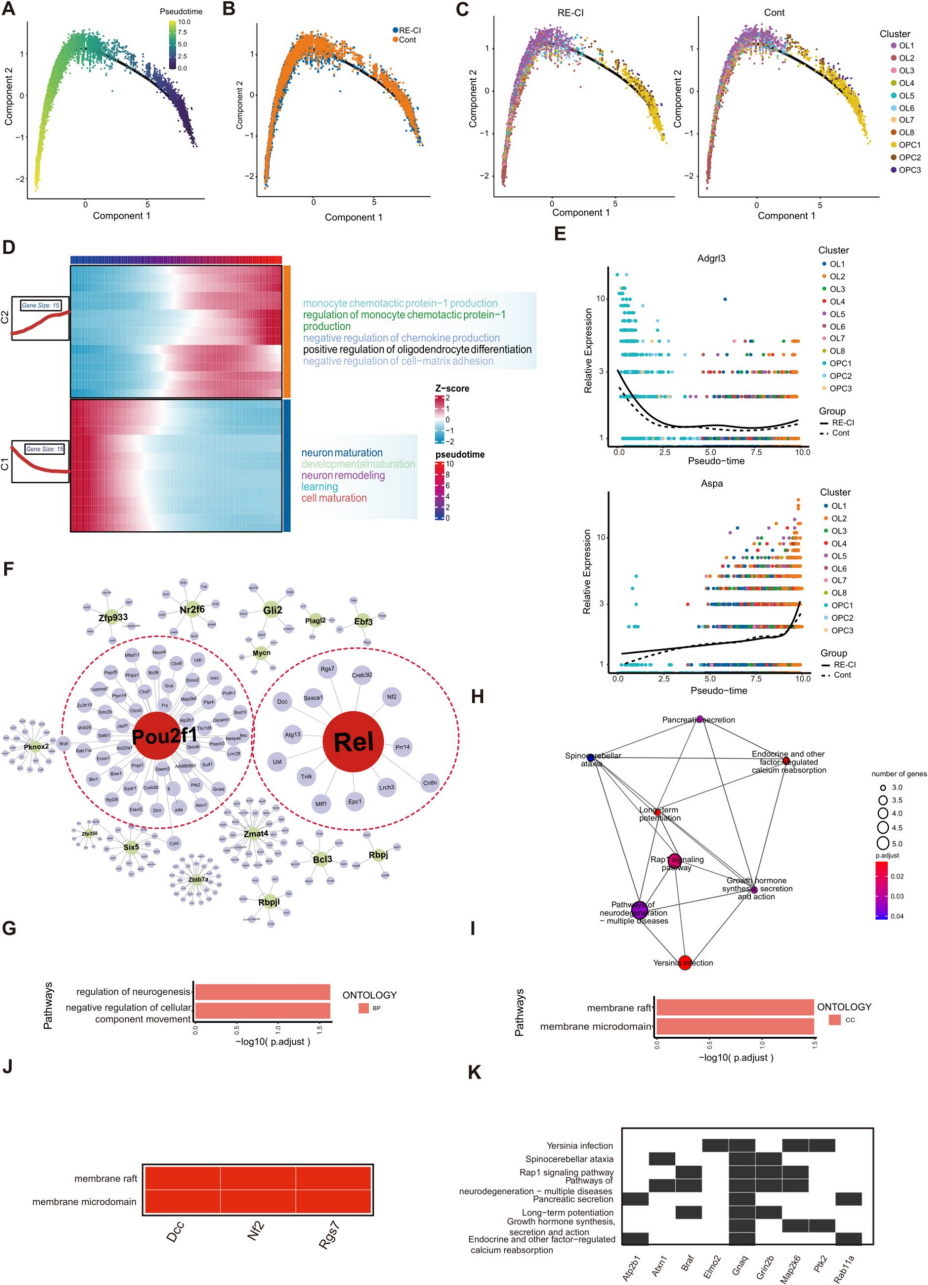
Trajectory analysis of oligodendrocyte progenitor cells and oligodendrocytes in adult epileptic cognitive impairment. **A**) Reverse chronological trajectories of oligodendrocyte progenitors and oligodendrocytes, horizontal coordinate is pseudotime, vertical coordinate is density (density, cells analyzed by all parameters are shown) of cell numbers at different time points. Different colors indicate the density distribution of different cell types with respect to pseudotime. **B**) Intergroup distribution density of oligodendrocyte progenitors and oligodendrocytes along the pseudotemporal trajectory, color represents the group. **C**) Distribution density after subdivision into subpopulations, the horizontal coordinate is pseudotime, the vertical coordinate is the density of the number of cells at different time points (density, shown by cells analyzed by all parameters). Different colors indicate the density distribution of different cell types with pseudo time. **D**) Demonstrate the dynamics of gene expression with pseudo time, the horizontal coordinate from left to right represents the time from small to large, the differential genes are classified into 2 clusters, and the representative enriched pathways of each group are shown in the vertical coordinate, and each point indicates the expression of the specified genes at the specified pseudo time (the mean). **E**) Demonstrate the expression of the genes with the pseudo time changes in the expression of the genes. Horizontal coordinates from left to right are representative time from small to large, vertical coordinates are the expression of genes, and different colors indicate different cell types. **F**) TOP16 transcription factors interacting with target genes based on the protein level, in which we focus on Pou2f1 and Rel, data from String database. **G**) GO database analysis based on molecules interacting in the presence of Pou2f1. **I**–**J**) GO database analysis of molecules based on the presence of interactions of Rel. H–K) KEGG database analysis of molecules based on the presence of interactions of Pou2f1

Analysis of the proposed temporal trajectory of OPCs to OLs showed an overall trend of differentiation from OPCs to OLs, with OPCs represent the initial state in pseudotime, while OLs occupy the terminal differentiation state (Fig. 4A, C). In terms of cell distribution density, the overall OL density in RE-CI corresponded to the number of cells in Fig. 1, both of which were increasing (Fig. 4B). To explore the biological basis of cell differentiation trends, we conducted enrichment analysis on differential molecules at the start and end points, respectively. The OL end showed positive regulation of the OL differentiation pathway, indicating that differentially expressed gene sets at the mature end of OL differentiation promotes the differentiation and maturation of OLs. The OPC end was enriched for cell maturation, and developmental maturation pathways, and that part of the gene collection promoted the differentiation of OPCs (Fig. 4D). We analyzed the differentially expressed genes based on the changes in the proposed temporal trajectories and found that Adgrl3 was highly expressed in OPCs. The decrease in Adgrl3 expression seemed to be a marker of OPC maturation, whereas the high expression of Aspa in the OL subset can be used as a marker for the formation of mature OLs (Fig. 4E).

We selected the TOP16 transcription factors with large intergroup differences to visualize their target genes and performed pathway enrichment analysis (Fig. 4F and TableS6). The results showed that the target genes of Pou2f1 and Rel were enriched in pathways related to cognition. First, the target genes of Pou2f1 were significantly correlated with the regulation of neurogenesis, which was highly correlated with a variety of neurodegenerative diseases, thus, suggesting an important role for the transcription factor Pou2f1 in epileptic cognitive disorders (Fig. 4G). In ischemic stroke, Pou2f1 is involved in neural repair as a transcription factor in OLs [32]. Analysis of the KEGG database revealed that Pou2f1 target genes, such as Braf, Gnaq, and Grin2b, among others, are significantly associated with the LTP pathway and are significantly different in epileptic cognitive impairment (Fig. 4H, K). Rel target genes, including Dcc, Nf2, and Rgs7, are associated with lipid raft membranes and are likely involved in membrane protein transfer (Fig. 4I–K).

### Astrocytes Modulate Myelin Changes in Adult Epileptic Memory-Impaired Mice

**Fig. 5:**
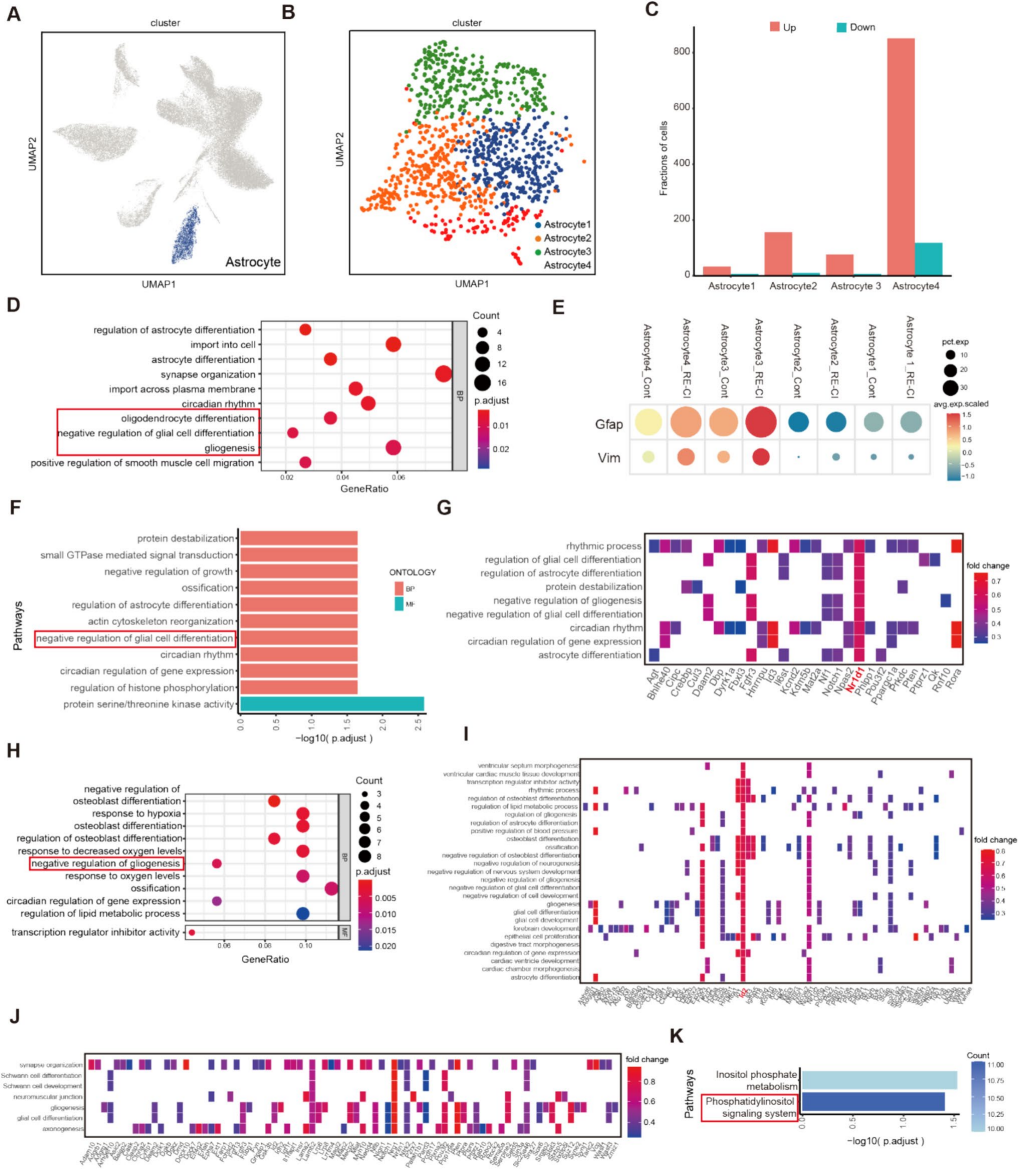
Astrocytes modulate myelin changes in adult epileptic memory-impaired mice. **A**) UMAP plot of astrocytes subjected Astrocytes to Uniform Mobility Approximation and Projection (UMAP) analysis. **B**) Astrocytes were divided into 4 subgroups. **C**) Bar chart of the results of the intergroup DEG differential expression analysis of Astrocytes subgroups, with the horizontal axis representing the 4 subgroups, the vertical axis representing the number of DEGs, and the color representing the group. **D**) Upregulation of Astrocytes subgroups for GO BP enrichment analysis, the size of the circle represents the number of genes enriched to, the color represents the *P*-value. **E**) Bubble plot of intergroup differential expression analysis of GFAP and VIM among subgroups of Astrocyte1-4. **F**) Analysis of upregulated GO enrichment in subgroups of Astrocyte2, showing the two parts of BP and MF, the longer bar represents the more significant difference, and the vertical axis is the GO Term. **G**, **I**, **J**) Inter-subgroup upregulation of GO network showing the key molecules significantly involved in the signaling pathway (key molecules are labeled, G-Astrocyte2, I-Astrocyte3). **H**) Upregulation GO enrichment analysis between subgroups of Astrocyte2, showing both parts of BP and MF, the darker columns represent the more significant differences, and the vertical axis is the GO Term. **K**) Inter-subgroup upregulation KEGG enrichment analysis between subgroups of Astrocytes, the longer columns represent the more significant results

The protein network database showed that the MBP transcriptional repressor Lingo1 correlates with GFAP. To verify this correlation, we analyzed the astroglial data in detail, and the downscaling clustering showed four classes of astrocytes: Astrocyte1, Astrocyte2, Astrocyte3, and Astrocyte4 (Fig. 5A–B and TableS4). Astrocytes are enriched in epileptic memory disorders OL differentiation, negative regulation of glial cell differentiation, and gliogenesis in the GO pathway, suggesting overall negative regulation of OL differentiation by astrocytes and inhibition of myelin regeneration (Fig. 5D).

The accepted classification of astrocytes is neurotoxic A1 phenotype astrocytes and neuroprotective A2 phenotype astrocytes [33]. We used the traditional activation form to classify the four types of astrocytes. The Astrocyte1-4 subpopulations all showed GFAP enrichment, with Astrocyte3 and Astrocyte4 being the most activated. The classification results showed that Astrocyte4 was probably a protective astrocyte, while Astrocyte3 was the neurotoxic type (Fig. 5E). Select three subgroups of astrocytes with relatively high levels of DEGs for further analysis (Fig. 5C). Astrocyte2 and Astrocyte3 upregulated molecules were enriched in GO pathways such as negative regulation of glial cell differentiation and negative regulation of gliogenesis (Fig. 5F, H), in which Nr1d1 and Id2 play a role. Id2 is a transcriptional regulator targeting MBP and is significantly elevated in demyelination-associated neurodegenerative diseases [34]. Id2 inhibits OL differentiation and myelin regeneration (Fig. 5I) [35, 36]. However, Nr1d1 was shown to be downregulated in astrocyte-neuron associations, inducing pro-inflammation and neuronal death in astrocytes [37]. Thus, the high Nr1d1 expression observed here may suggest a protective role for Astrocyte3 (Fig. 5G).

Schwann cells are a population of cells wrapped around an axon, of which the myelin sheath is a part, and consists of a cytosolic membrane. Astrocyte4 is enriched for synapse organization, gliogenesis, Schwann cell development, neuromuscular junctions, Schwann cell differentiation, glial cell differentiation, and the GO term for axonogenesis. Thus, Astrocyte4 is closely related to the promotion of myelin regeneration and OL differentiation (Fig. 5J). KEGG results showed that the Astrocyte4 subpopulation could promote lipid metabolism and myelin production (Fig. 5K).

### Microglia Play a Bidirectional Regulatory Role in Adult Myelin Regeneration Process

**Fig. 6:**
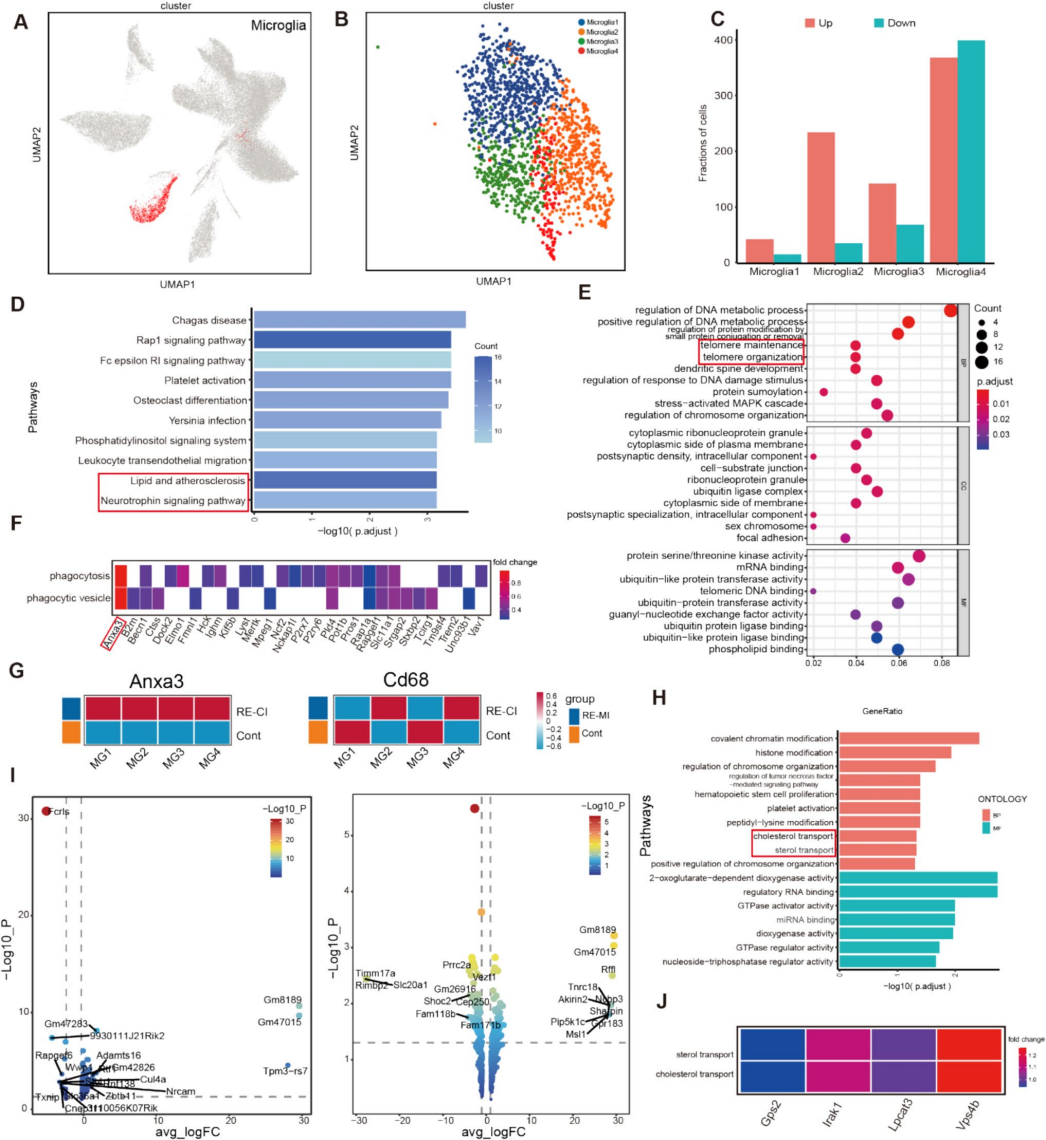
Bidirectional regulatory role of microglia in adult myelin regeneration. **A**) UMAP plot of microglia. **B**) microglia are divided into 4 subgroups. **C**) Bar graph of the results of intergroup DEG difference analysis of Microglia subgroups, the horizontal axis represents the 4 subgroups, the vertical axis represents the number of DEGs, and the color represents the group. **D**) Intergroup upregulation of KEGG enrichment analysis of Microglia, the longer the bar represents the more significant result. **E**) Intergroup upregulation of GO of Microglia subgroups enrichment analysis, the size of the circle indicates the number of genes enriched to, and the color represents the *P*-value. **F**, **J**) Inter-subgroup upregulation of GO network showed that the key molecules were significantly involved in the signaling pathway (key molecules have been labeled, F-Microglia, J-Microglia2). **G**) Differential expression heatmap of Cd68 versus Anxa3 among the Microglia1-4 subgroups. **H**) GO enrichment analysis of the upregulation of Microglia4 subgroups, the longer the columns represent the more significant results. **I**) MicrogliaVolcano plot of the inter-subgroup differential analysis of OPC2. The horizontal coordinate is the logFC value of the average expression, the vertical coordinate is the absolute value of log10(P) or the difference between pct.1 and pct.2. pct.1: the percentage of cells in which the gene was detected in the first group; pct.2: the percentage of cells in which the gene was detected in the second group. The names of the genes in the top10 are shown (sorted by avg_logFC value); the size and color of the points when the vertical coordinate is log10(P) represent log10(P), and the size of the points when the vertical coordinate is pct represent pct size and the color represents log10(P)

Microglia are the first cell population to respond to pathological injury in the brain and play a double-edged role in the CNS, classically categorized as M1 pro-inflammatory M2 neuroprotective microglia [33]. Single-cell dimensionality-decreasing clustering classified microglia into four subpopulations: Microglia1-4 (Fig. 6A–B and TableS4), and according to CD68 distinguishes activated microglia occurring in subpopulations 2 and 4, which were the focuses of our attention (Fig. 6G).

Microglia4 had a higher number of DEGs, both up- and downregulated, than microglia1, microglia 2, microglia 3 had a significantly higher number of upregulated genes than downregulated genes (Fig. 6C). To validate the effect of epileptic cognitive impairment on microglia, microglia KEGG enrichment analysis focused on processes such as"lipid and atherosclerosis"and"neurotrophin signaling pathway"(Fig. 6D). This is consistent with our previous finding whereby mic can phagocytose more than the produced myelin, and thus, modify the myelin sheath, preventing the appearance of myelin hyperplasia, as well as preventing demyelination and maintaining the existing myelin sheath [13]. Microglia promote myelin regeneration by phagocytosing debris produced by demyelination [38]. Therefore, we searched for key molecules related to phagocytosis and chose the terms"phagocytosis"and"phagocytic vesicle"within GO terms to observe the molecular characterization therein, and found that Anxa3 differed significantly between the two (Fig. 6F). Enrichment analysis of the expression level of Anxa3 among different subgroups. Microglia2 enrichment analysis showed the presence of"telomere maintenance"and"telomere organization". Telomeres are inextricably linked to cellular senescence, which leads to a failure of myelin regeneration because of decreased phagocytosis by aging microglia and ultimately reduces the removal of myelin debris (Fig. 6E). Among the differentially expressed genes in Microglia2, Fcrls was significantly downregulated, which is a mic-specific gene, and its high expression enhances mic homeostasis. Here, it seems to suggest that the homeostasis of M2 microglia is disrupted and may hinder myelin regeneration (Fig. 6H).

However, we found GO terms such as “cholesterol transport” and “sterol transport” in Microglia4, suggesting that the M4 subpopulation may be involved in promoting lipid metabolism processes that favor myelin regeneration (Fig. 6I). Microglia4 upregulates molecules such as Rffl, Tnrc18, Akirin2, Pip5k1c, and Msl1, which may be directly or indirectly related to myelin regeneration (Fig. 6H). We searched for molecules that interacted between"cholesterol transport"and"sterol transport"and found Vps4b, Irak1, Lpcat3, and Gps2. Among them, Vps4b regulates neuronal death, Irak1 is implicated in neuroinflammation involving microglia, Lpcat3 affects lipid metabolism and microglia numbers in the brain [39], and Gps2 plays a role involving macrophage inflammatory activation (Fig. 6J) [40].

### Communication Links Between Three Glial Cell Types in Cognitive Impairment

CellChat helps to identify complex signaling communication networks and establishes complex signal transduction and receptor-ligand information between various cell populations [41]. We chose CellChat to analyze the link between myelin regeneration and intercellular interactions in memory disorders.

**Fig. 7:**
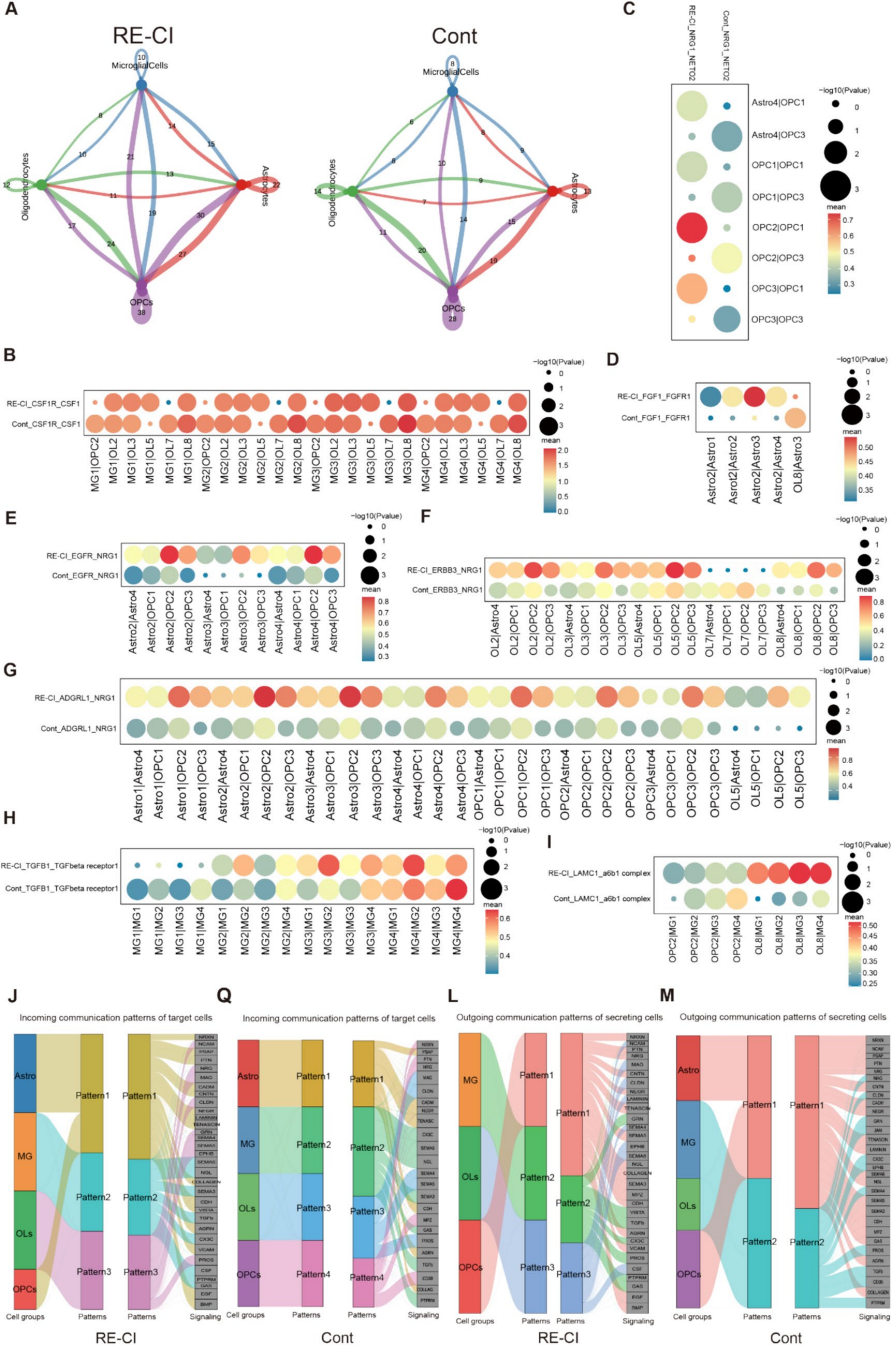
Communication links between myelin regeneration-associated glial cells in adult epileptic memory disorders. **A**) Shows the interactions between all cell populations between groups, the numbers on the lines indicate the number of interaction pairs, and the thickness of the lines is proportional to the number of interaction pairs. **B**–**I**) Shows the differential receptor pairs between two cell types, the size of the circle represents the significance of the interaction, and the color of the circle represents the strength of the interaction (gene expression). **J**–**M**) Coordinated functioning of multiple cell populations and signaling pathways, the left panel shows 4 cell types divided into different patterns, the right panel shows the key input or output signals of each pattern

First, Cont and RE-CI intergroup cell subpopulation interaction pair counts revealed an increase in the number of interaction pairs between OPCs and astrocytes, microglial, and OLs, showing that OPCs play an important function in overall signaling communication (Fig. 7A). Among all the receptor-ligand pairs, we were interested in eight pairs of receptor ligands. The receptor-ligand pairs found to be associated with OL maturation and differentiation, such as ADGRL1-NRG1, EGFR-NRG1, ERBB3-NRG1, FGF1-FGFR1, and NRG1-NETO2; This reflects the fact that pairs of Astrocytes, OLs and OPCs themselves can promote OPC differentiation, suggesting that cell-to-cell interactions concerning myelin regeneration-associated pathways do exist in adult epileptic memory disorders. Signaling promotes OPC maturation indirectly promoting myelinating OLs. There is also a part of the interaction pairs which affects microglia activation, apoptosis, and phagocytosis, such as LAMC1-a6b1 complex, and TGFB1-TGFbeta receptor1 (Fig. 7B–I). CSF1R inhibitors can clear microglia and the molecule promotes microglia growth, but the results show that after epileptic memory disorders, OL7 may inhibit microglial cell growth and affect its role. OL8 is highly expressed in the RE-CI group and the LAMC1-a6b1 complex may affect the migration and aggregation of microglial cell subsets (Fig. 7B–I).

CellChat enriched 30 differential signaling pathways, four of which may be related to myelin regeneration, including the PTN signaling pathway network, CNTN signaling pathway network, EPHB signaling pathway network, and JAM signaling pathway network. PTN as an autocrine factor can promote OL differentiation and myelin repair. PTPRZ inhibits proliferation and promotes maturation by binding to CNTN1 expressed on the surface of OPCs. EPHB directs peripheral neural regeneration through SOX2-dependent sorting of Schwann cells. JAM2 inhibits myelin sheath production in dendrites.

In addition to communication between individual cells, we also considered interactions between cell populations. We first assigned patterns to the input–output inter-communication subpopulations based on differential genes, then screened the marker genes for each pattern, and subsequently enriched the respective signaling pathways into the patterns, thus identifying the links between the subpopulations and the signaling pathways (Figure S3). Based on the above four signaling pathways, we picked the corresponding signal emitters and accessors. This analysis accessed four and three patterns of signaling efferent and two and three patterns of signaling efferent (Fig. 7J–M). We compared the differences in signaling afferent patterns among the three glial cells between groups. A subpopulation of OPCs was found to play a signaling role in the Cont group with Pattern4, which included the PTN signaling pathway. However, in the RE-CI group, OPCs shared Pattern1 with Astrocytes to exert signaling, which also included the PTN signaling pathway (Fig. 7L–M). And that astrocytes may have indirectly promoted myelin regeneration through the PTN signaling pathway by increasing the differentiation of OPCs. Focusing on the efferent patterns between groups, astrocytes shared pattern1 with OPCs in the Cont group, while OPCs were predominantly pattern1 in RE-CI (Fig. 7 G–Q). Microglia directed neural regeneration of OLs through EPHB signaling in the Cont group, but only OLs themselves were present in the RE-CI group, which may have contributed to the electron microscopy results of myelin sheath thickening because microglia can prune myelin during myelin generation (Fig. 7L–M).

### Promoting Myelin Regeneration Facilitates Cognitive Impairment

**Fig. 8:**
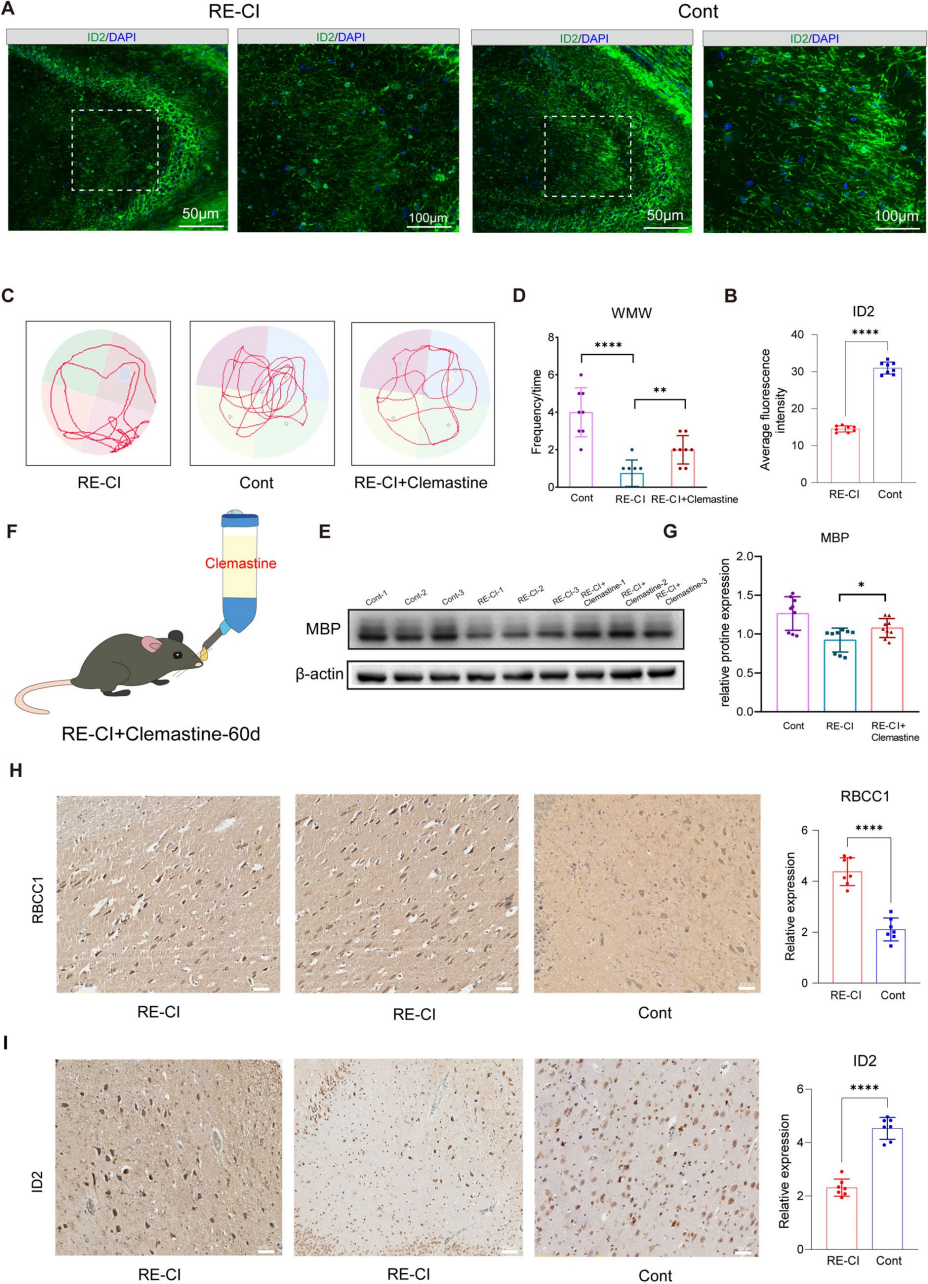
Promoting myelin regeneration favors cognitive impairment. **A**) ID2 staining of brain sections of RE-CI and Cont mice, the field of view was taken from 20 × and 40 ×. **B**) This is the statistics of the mean fluorescence intensity, using the unpaired *t*-test of the two groups. ****P < 0.001. **C**) Water maze trajectory graphs with RE-CI and Cont after 60D of Clemastine treatment. **D**) Statistics of water maze results between RE-CI, Cont and RE-CI + Clemastine groups using unpaired *t*-test for both groups, ****P < 0.001. **E**) Graphical representation of MBP protein expression in mouse hippocampus between RE-CI, Cont and RE-CI + Clemastine groups. The original image is shown in Figure S5-6. **F**) Graphical representation of RE-CI mice treated with Clemastine for 60D, see Materials and Methods for details. **G**) Statistics of MBP expression based on Figure E, using unpaired t-test for both groups, *P < 0.05. H) Effects of OLs, OPCs, Microglia, and Astrocytes on Myelin Regeneration Mechanism diagram. **H**–**I**) Immunohistochemical staining of RB1CC1 with ID2 in temporal lobe tissue of RE-CI patients and Cont patients is illustrated. Statistical illustrations are shown on the right

In our previous analysis, we found that OL myelin regeneration is closely related to OPC differentiation and maturation. RB1CC1 + is a major marker of OL differentiation and maturation, and immunofluorescence staining showed that the average fluorescence intensity of RB1CC1 + OLs were enhanced in the hippocampus of RE-CI mice, which indicated that myelin regeneration existed in these animals, thus, corroborating the previous molecular argumentation (Fig. 3O). ID2 can be targeted directly to Olig2 to negatively regulate OL differentiation. The mean fluorescence intensity of ID2 in the hippocampal region was significantly decreased in RE-CI mice, and the results all clearly showed that cognitive deficits in RE-CI mice were associated with the maturation of OPC differentiation (Fig. 8A–B). Belcin promotes phagocytosis of Microglia, and it has been shown that Microglia phagocytosis of myelin debris to release myelin assists in myelin regeneration, which is helpful to reduce amyloid accumulation and prevent cognitive impairment in Alzheimer's disease. Furthermore, in our study, a significant increase in the mean fluorescence intensity of belcin in DRE suggests that there is activation of autophagy, which contributes to the clearance of myelin debris and promotes myelin regeneration (Figure S4A). The expression intensity of Tns3 was significantly decreased in DRE, and we suggest that myelin regeneration in DRE is not related to the fact that the differentiation of OPCs is not dependent upon Olig2 (Figure S4B).

The regulation of differentiation in OLs may be due to the fact that Mir219 present in OLs targets SOX6 in OPCs and thus plays a role in promoting OPC differentiation (Figs. 2–3).

It has been shown in the literature that Clemastine can be used to treat cognitive impairment by promoting the differentiation of OPCs and the production of myelinated OLs. We took the hippocampal tissues of RE-CI mice for validation after feeding them 60D, according to the dosage in a previous publication by Jing-Fei Chen et al. (Fig. 8F), and found that hippocampal regional MBP protein expression levels were significantly elevated compared to RE-CI, but did not reach the level of Cont, thus, demonstrating that pro-OPC differentiation myelin therapy was effective for regeneration (Fig. 8E, G). Next, we performed water maze experiments to detect the memory capacity of mice in the treatment group and the control group at the same time. We found that the cognitive impairment of mice in the treatment group was significantly improved, and that the time and number of times they stayed at the platform were significantly increased when compared to the RE-CI mice (*P* < 0.05 but did not reach the normal control level) (Fig. 8C–D). This demonstrated that the mechanism of myelin regeneration in cognitive impairment in adult DRE was closely related to OPC differentiation. We also verified the samples in the epilepsy specimen bank of the neurosurgery department of Tangdu Hospital and showed that RB1CC1 expression was significantly elevated in patients with definite cognitive impairment. ID2 expression was also significantly decreased, which was in agreement with the results from our immunofluorescence studies in mice (Fig. 8H–J and TableS7). Therefore, it can be seen that myelin regeneration in cognitive impairment in adult DRE is closely related to OPC differentiation. Promotion of OPC differentiation significantly improved the memory problems in cognitively impaired mice with DRE, suggesting that DRE produces myelin sheaths or weak or loss of axon function despite the presence of myelin regeneration. Such a change also involves a booster for Microglia and astrocytes to work together to promote myelin regeneration in adult RE-CI mice.

## Discussion

Our study reveals the role of OPCs、OLs、Astrocytes and Microglia in the process of myelin regeneration in adult drug-resistant epileptic memory disorders, where myelin regeneration would favor memory improvement. OPCs differentiate and mature into OLs to form myelin, which is wrapped around the axon to protect it and facilitate the spatial transfer of macromolecules in the axon [42]. Overall, we have screened in detail the molecular basis for the positive or negative regulation of myelin regeneration in the three major glial cells and their relevant pathways enriched for the subsequent basic research on myelin regeneration in adult epileptic memory disorders. We also found that myelin regeneration, which is present in adult memory disorders, did not play a specific role in the improvement of memory disorders, possibly due to axonal degeneration. The molecular mechanisms of OLs themselvesf are altered when they are affected, leading to impaired support of OLs to the axon. We prompted a significant therapeutic effect of myelin regeneration using drugs promoting OPC differentiation, and further enhanced expression of molecules associated with myelin regeneration, thus revealing a possible molecular basis for myelin regeneration in adult memory disorders.

### OLs Are the Basis for Myelin Formation, and Alterations at the Molecular Level of OPCs Are Sufficient to Affect OLs and Myelin Production

OLs is a myelin-forming cell, and myelin regeneration in drug-resistant epileptic memory deficits did not ameliorate memory deficits caused by what we consider demyelination, and there is evidence that Myo5a and Pbrm1 are associated with peripheral axons, and that myelinated axonopathy greatly impairs the maintenance of memory is probably a large part of the reason for this. Previous evidence suggests that OPCs is closely related to the production of OLs myelin. A large number of OPCs that failed to differentiate normally into OLs were found in demyelinated regions in patients with multiple sclerosis [43]. Memory deficits are also common in patients with multiple sclerosis and are closely related to reduced hippocampal activation [44]. There is support in the literature for the involvement of OPCs in myelin damage and regenerative effects in multiple sclerosis [45]. In epileptic memory disorders we revealed that OPC1 and 3 subpopulations are homeostatic subpopulations and OPC2 undergoes a greater stress response in the pathological setting of epileptic memory disorders. The upregulated ErbB signaling pathway is involved in OPC2, for example, where the upregulated keystone molecule Nrg1 serves as a neuromodulatory protein that promotes neurodevelopment. OPC2 also involves upregulated molecules such as Cd9, and Tns3 of which Cd9 is involved in the maintenance of the overall myelin structure and promotes myelin protection [29], and Tns3 is closely related to the promotion of the differentiation of OPCs into OLs [27]. OPC3 is in a greater proliferation more state as well as proliferation and differentiation, and promotes the generation of OLs, and is involved in pathways related to mitotic cytokinesis, cytoskeleton-dependent cytokinesis and other pathways. Cep55 plays a role in cytoplasmic division, which is the best evidence of enhanced proliferation and differentiation of OPC3 [30].

### Two-Sided Effects of Astrocytes on Myelin Plasticity

Previous studies have shown that Astrocytes are bifacial for myelin regeneration, and Astrocytes can secrete some cytokine-like substances, which in turn induces OPC maturation and myelin regeneration [46]. Astrocytes represent the second population of phagocytic cells in the nervous system as distinct from Microglia. Under healthy conditions, they phagocytizes OLs, OPCs and unwanted myelin sheaths and promote the formation of myelin. In pathological conditions, however, they lead to excessive phagocytosis or debris that are not well cleared, leading to inflammation and inhibition of myelin regeneration [47]. The String database shows a strong correlation between Lingo1 and GFAP proteins, and GFAP, a marker of Astrocyte activation, suggesting a role for Astrocytes in myelin regeneration in drug-resistant epileptic memory disorders. While the analysis of our results revealed that Astrocytes as a whole play a negative regulatory role in myelin regeneration and repair. High expression of Id2 mediated by Astrocyte2 regulates demyelination-related activities, inhibiting oligodendrocyte differentiation and myelin regeneration. Nr1d1 triggers the protective mechanism of Astrocyte3, where GeneCards showed that Nr1d1 inhibits Microglia activation, promotes steroid biosynthesis, undergoes ubiquitination limiting the inflammatory response. The String database shows that Nr1d1 interacts with Ncor1, arntl, npas2, per2, and other proteins, and is involved in transcriptional regulation of repression and activation, and regulation of body metabolism. Nuclear receptor corepressor 1 (NCoR1) is involved in metabolism and inflammation, and its decreased expression induces inflammation in alcohol-related liver disease (ALD). In alcohol-associated liver disease (ALD), decreased expression of NCoR1 induces inflammation [48]. Per2 clearly can regulate the DRD1-PKA-CREB signaling pathway thereby affecting spatial memory in mice [49]. Arntl, npas2 are two circadian rhythm-related proteins, and dysregulation of circadian rhythms is closely related to memory deficits therefore, Nr1d1 may modulate the rhythmic proteins of organisms or memory-directly related pathways to play a protective role.

### Two-Sided Effects of Microglia on Myelin Plasticity

There is support in the literature that when microglia receive external stimuli, they cause disruption of glycolysis and mitochondrial dysfunction, leading to the expression and secretion of pro-inflammatory factors, which ultimately cause an inflammatory response [50]. There is clear evidence that microglia activation is enhanced during demyelination, which does not occur in Astrocytes and OPCs, suggesting that demyelination is accompanied by an inflammatory response and phagocytosis of myelin fragments by Microglia, and that an inflammatory response may be the main culprit of demyelination [51]. Memory transmission depends on potential changes on axons. However, activated Microglia can phagocytose synapses and affect signaling [52]. Microglia phagocytosis has two parts. Microglia phagocytosis of myelin and myelin debris reduces inflammation, promotes regeneration of myelin, and modifies excessive myelin to prevent hyperplasia, which is reflected in our KEGG enrichment results. Microglia phagocytosis and digestion of myelin debris generates new myelin for re-utilization. Downregulation of Fcrls may be responsible for demyelination. Downregulation of Fcrls reflects the disruption of Microglia homeostasis, and Anxa3 promotes inflammation to induce myelin detachment. Upregulation of Rffl, Tnrc18, Akirin2, Pip5k1c, and Msl1 molecules may be related to cholesterol metabolism and promotes the regeneration of myelin. Lpcat3 is also involved in lipid metabolism and may promote myelin regeneration. In conclusion the molecular changes in Microglia reflect the effects produced by Microglia's own phagocytosis and clearance on OLs myelin.

Of course there are some limitations in our study, we lack deeper mechanistic understanding of the cognitive impairment seen in drug-resistant epilepsy and did not derive a complete network regulating myelin regeneration in adults, but we provided a glial cell heterogeneity in the setting of cognitive impairment in adult drug-resistant epilepsy, which will provide a molecular basis for deeper understanding of the mechanism of myelin regeneration in adults.

Overall, in addition to the molecular basis related to demyelination normally found in drug-resistant epileptic memory disorders, the molecular basis for promoting myelin regeneration is present in the hippocampus of drug-resistant epileptic memory-disordered mice. The molecular mechanisms involved include cholesterol metabolism, and glial cell differentiation. Phagocytosis of Microglia promotes myelin reutilization, astroglutinin secretory proteins promote the cholesterol synthesis signaling pathway, and the OPCs secrete other substances enhancing their own differentiation to form myelin OLs, which together promote myelin regeneration under pathological conditions. Myelin regeneration did not ameliorate cognitive impairment, and we found that there is a molecular basis for inducing myelin peripheral axonopathy in OLs that impairs axon growth and ultimately does not cure cognitive impairment alone.

## Conclusions

We found that the problem of cognitive impairment in drug-resistant epileptic mice is due to altered myelin plasticity and that epilepsy induces changes in the cognitive environment mainly due to the promotion of OL differentiation. oL maturation induces pathological myelin regeneration which ultimately leads to cognitive impairment. snRNA-seq explains the molecular basis of myelin-associated cognitive impairment. We found that an increased number of OLs in drug-resistant epilepsy mice induced myelin regeneration e.g. OL5. but not all OL subpopulations were facilitated. For example, subgroups such as OL4, 6, and 7 inhibit myelin regeneration and play a protective role. oPC3 leads to an increase in myelin-producing OL5 through mitotic cytokinesis and cytoskeleton-dependent cytokinesis. RB1CC1 is significantly elevated in tissues from cognitively impaired patients. id2 is significantly decreased in tissues from cognitively impaired patients. RB1CC1 is significantly elevated in tissues from patients with cognitive impairment, and ID2 is significantly decreased in tissues from patients with cognitive impairment. Together, they promote myelin regeneration and affect cognitive impairment. Astrocyte4 and Microglia4 promote myelin regeneration indirectly by promoting OL differentiation and participating in lipid metabolism, respectively, while Microglia phagocytose myelin debris to release reusable myelin phospholipids. In conclusion, we found that the three glial cell types are closely related to the occurrence of myelin regeneration and jointly promote pathological myelin regeneration. Normal myelin recovery can be promoted by drug treatment. The treated mice were able to find the platform position relatively easily. MBP protein expression was significantly elevated in the hippocampus of treated mice. All of this evidence suggests that normal myelin regeneration contributes to cognitive impairment improvement, but pathological myelin regeneration impairs cognition.

## Supplementary Information

Below is the link to the electronic supplementary material.

Fig. 9 Supplementary figure 9

High resolution image (TIF 14.7 MB)

Fig. 10 Supplementary figure 10

High resolution image (TIF 14.7 MB)

Fig. 11 Supplementary figure 11

High resolution image (TIF 14.7 MB)

Fig. 12 Supplementary figure 12

High resolution image (TIF 14.7 MB)

Fig. 13 Supplementary figure 13

High resolution image (TIF 8.02 MB)

Fig. 14 Supplementary figure 14

High resolution image (TIF 9.77 MB)

ESM 7 (XLSX 8.88 KB)

ESM 8 (XLSX 8.86 KB)

ESM 9 (XLSX 9.27 KB)

ESM 10 (XLSX 15.8 KB)

ESM 11 (XLSX 9.04 KB)

ESM 12 (XLSX 12.1 KB)

ESM 13 (XLSX 11.3 KB)

## Data Availability

The study data can all be GSE241349 found at the GEO website.

## Abbreviations

***OPCs***: Oligodendrocyte precursor cells
***DRE***: Drug-resistant epilepsy
***RE-CI***: Drug-resistant epilepsy cognitive impairment
***LEV***: Levetiracetam
***OXC***: Oxcarbazepine
***LTG***: Lamotrigine
***VPA***: Valproate
***SGS***: Secondary generalized seizures
***CPS***: Complex partial seizures
***RTHAR***: Right anterior temporal lobe hippocampal amygdala resection
***EFR***: Epileptic Focal Resection
***FCD***: Focal cortical dysplasia
***g***: Gliosis
***DOC***: Donation of corpses
***TBI***: Traumatic brain injury
***LTL***: Left temporal lobe
***RTL***: Right temporal lobe
***IHC***: Immunohistochemistry
***F***: Female
***M***: Men
***w***: Week
***y***: Year
***GEO***: Gene Expression Omnibus
