# Supplementary figures and images for: SnRNA-seq Interprets Mechanisms by which Three Glial Cell Types Influence Myelin Regeneration in Adult Drug-Resistant Epilepsy-Related Cognitive Impairment
Source: Mol Neurobiol. 2025 Jul 17;62(11):14196–220. doi: 10.1007/s12035-025-05206-8 (PMC12511217; doi:10.1007/s12035-025-05206-8)

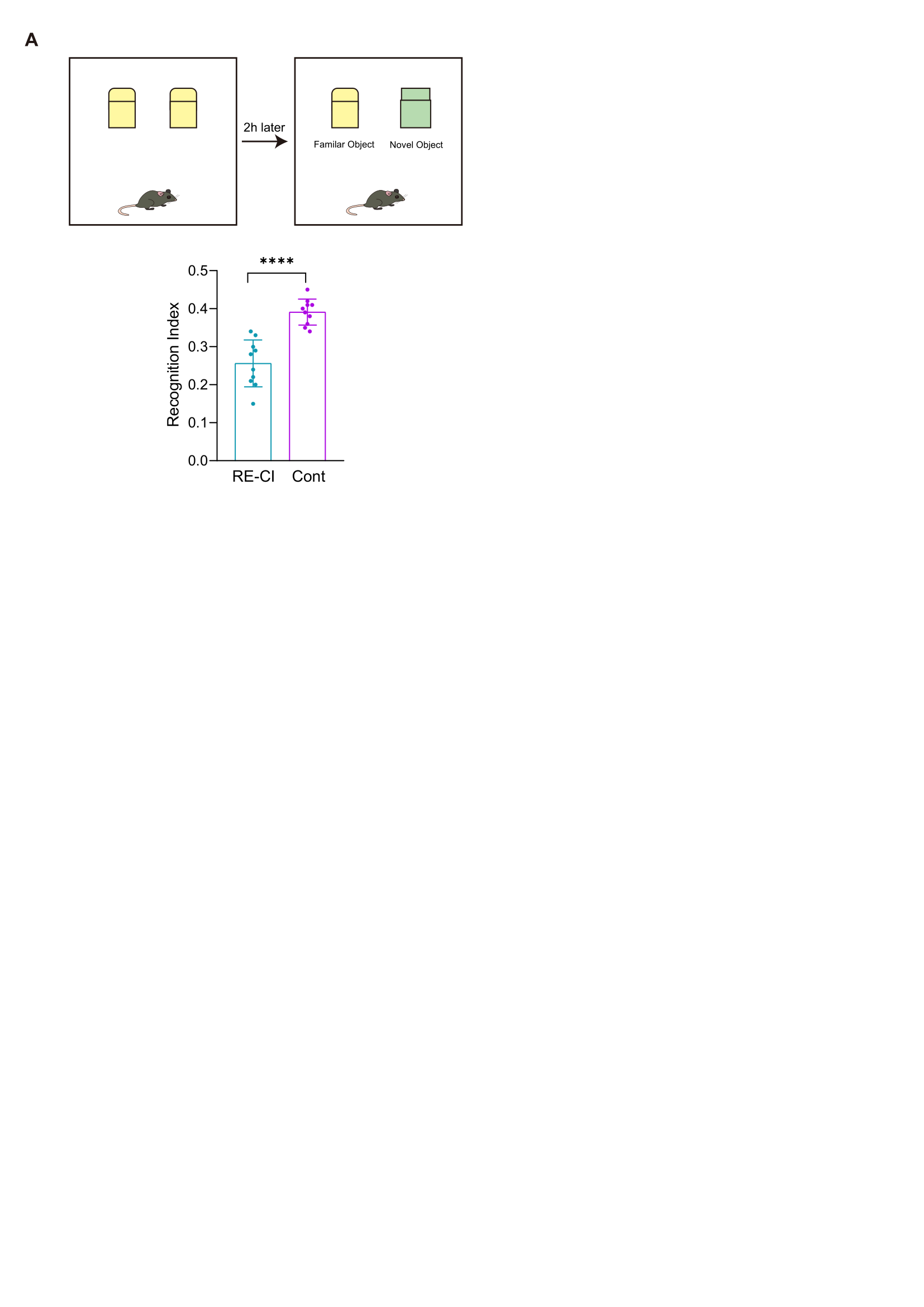

Supplement: Supplementary file 1 — Supplementary figure 9 [file 12035_2025_5206_Fig9_ESM.png]

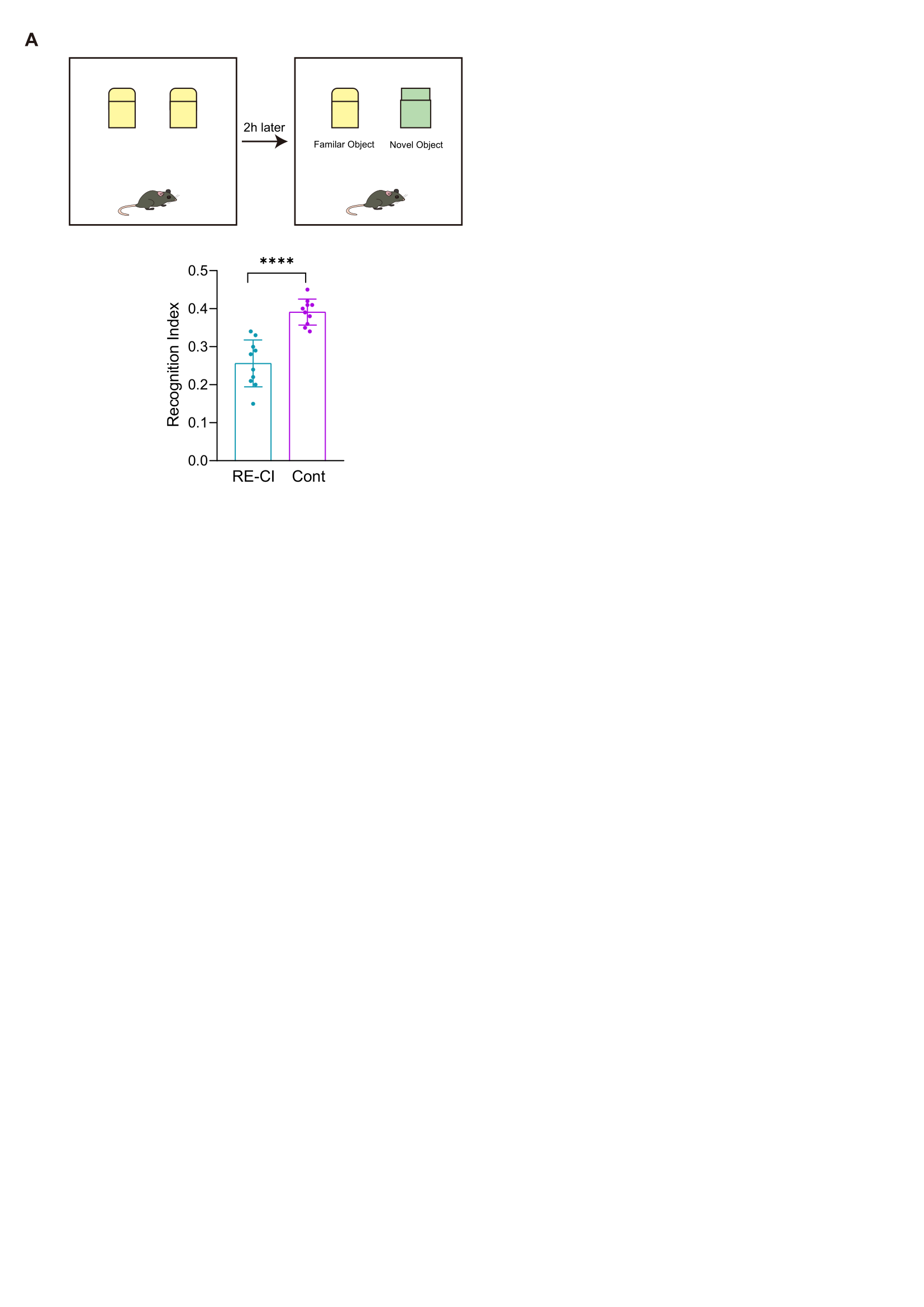

Supplement: Supplementary file 2 — High resolution image (TIF 14.7 MB) [file 12035_2025_5206_MOESM1_ESM.tiff]

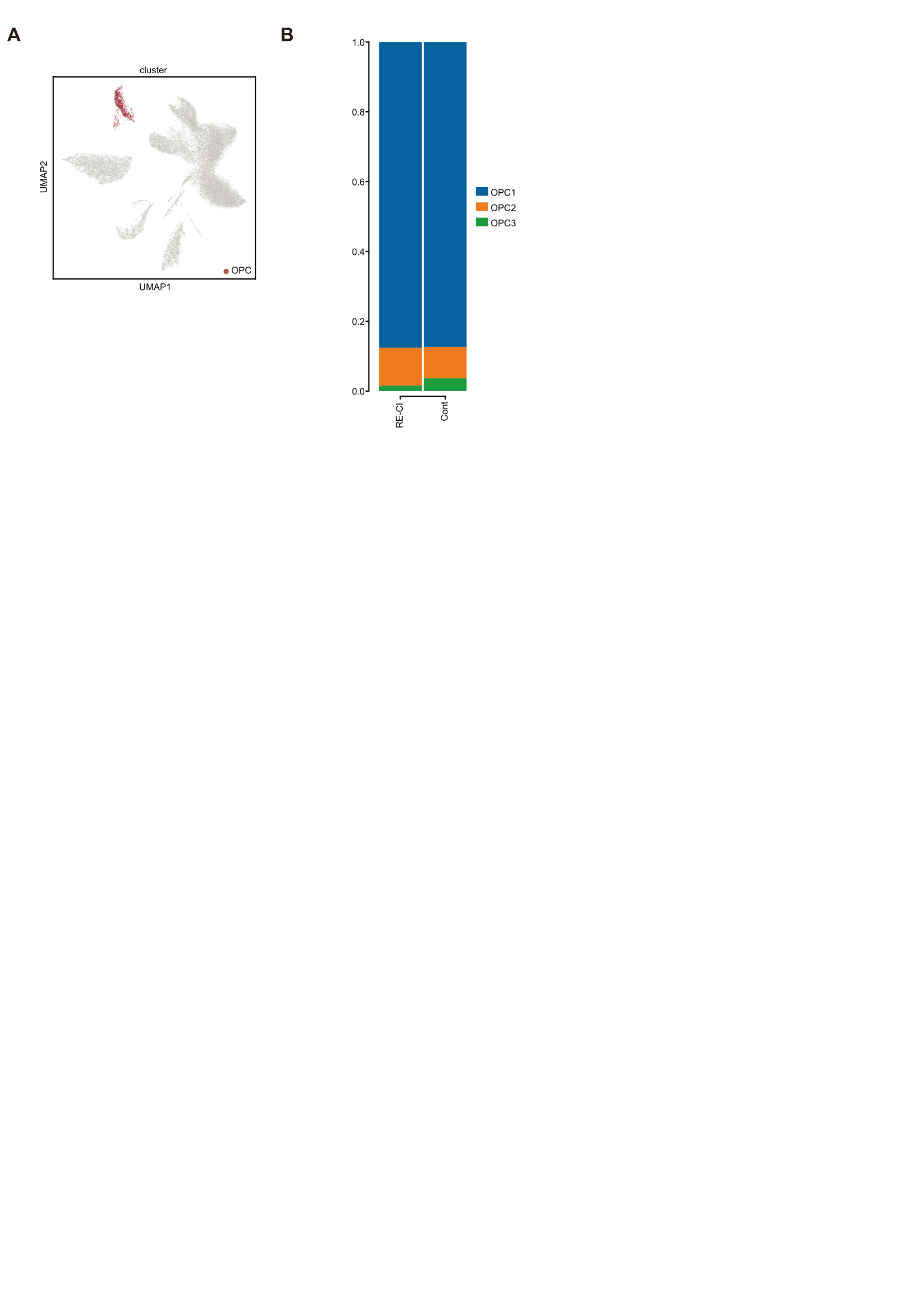

Supplement: Supplementary file 3 — Supplementary figure 10 [file 12035_2025_5206_Fig10_ESM.png]

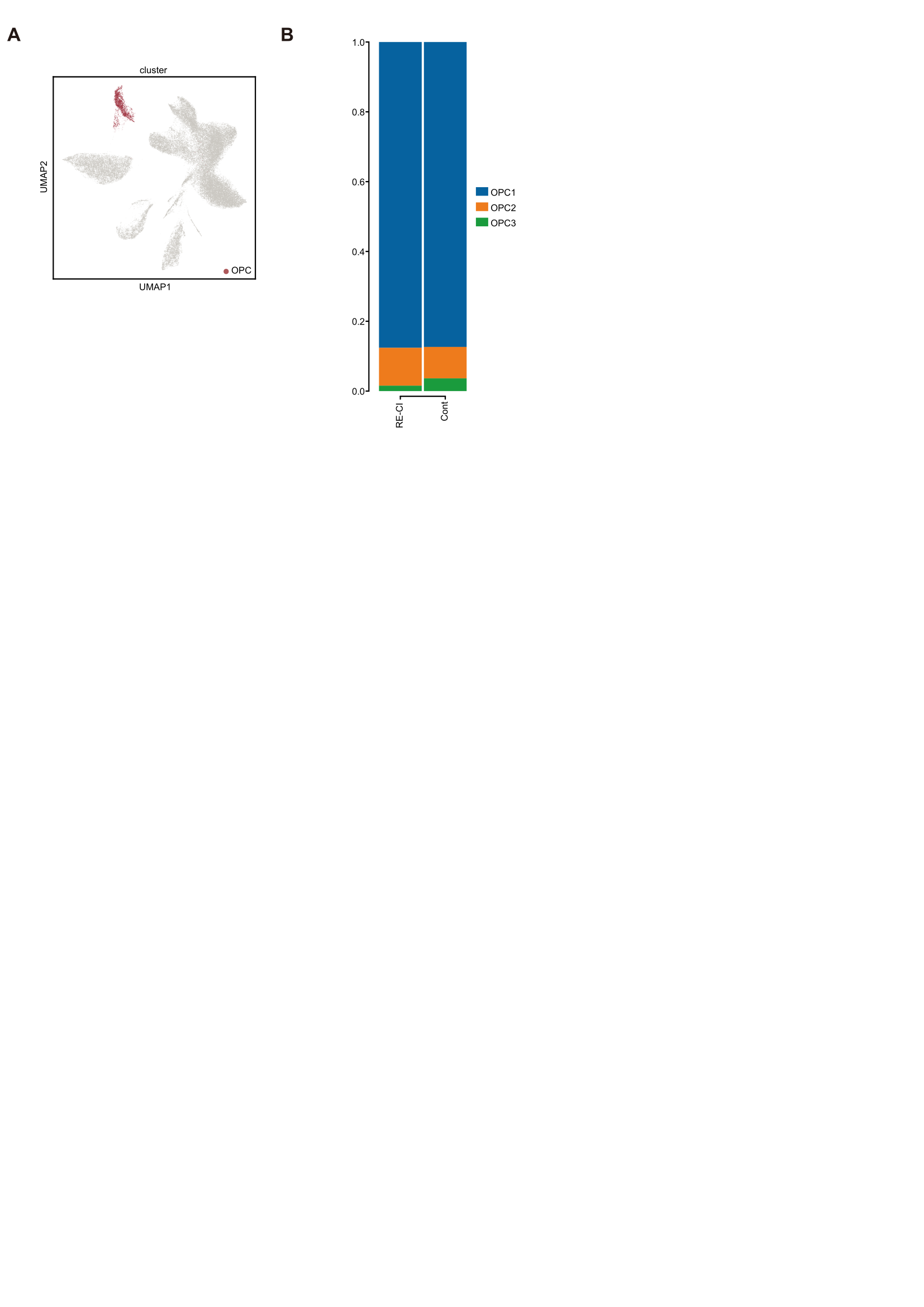

Supplement: Supplementary file 4 — High resolution image (TIF 14.7 MB) [file 12035_2025_5206_MOESM2_ESM.tiff]

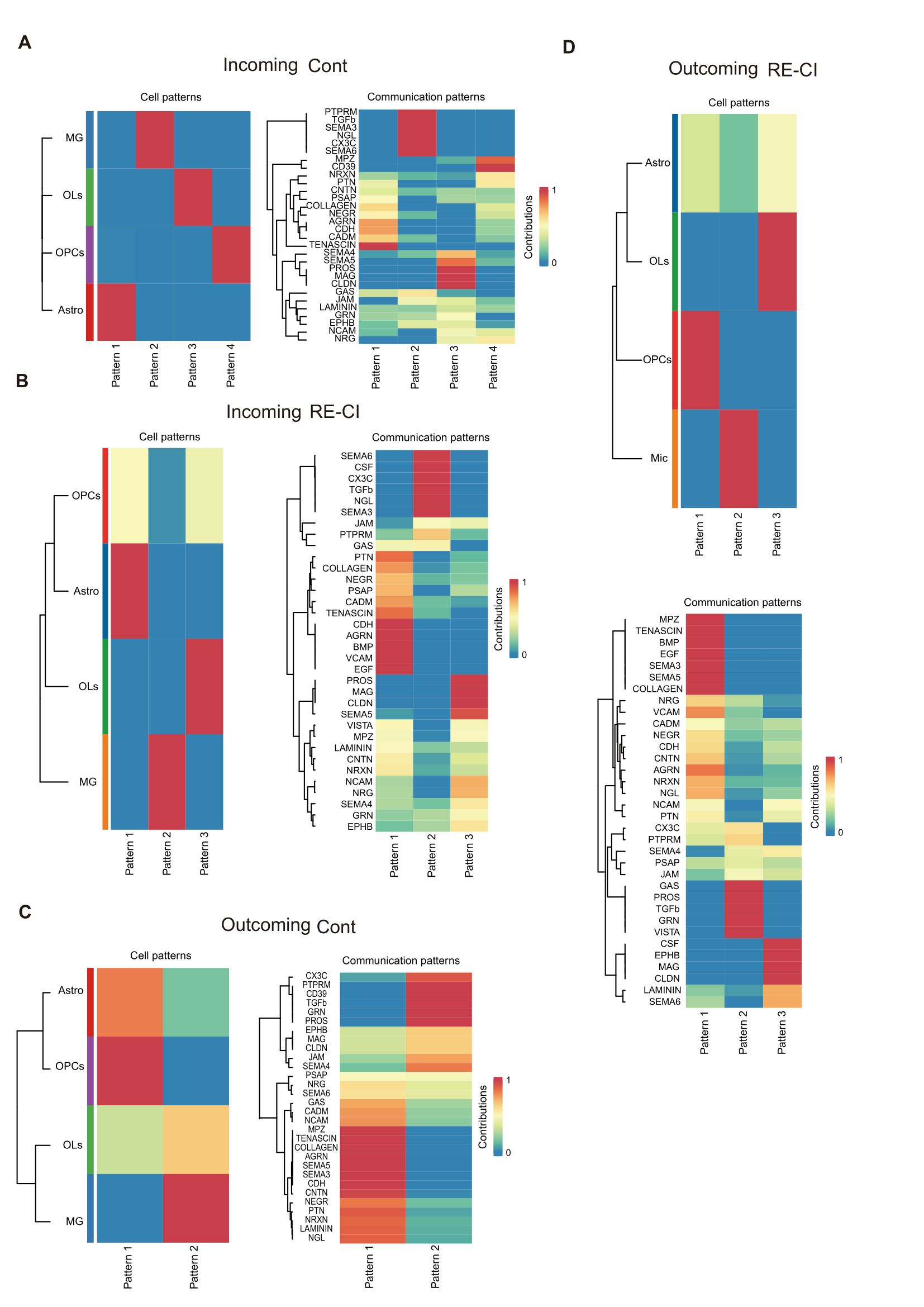

Supplement: Supplementary file 5 — Supplementary figure 11 [file 12035_2025_5206_Fig11_ESM.png]

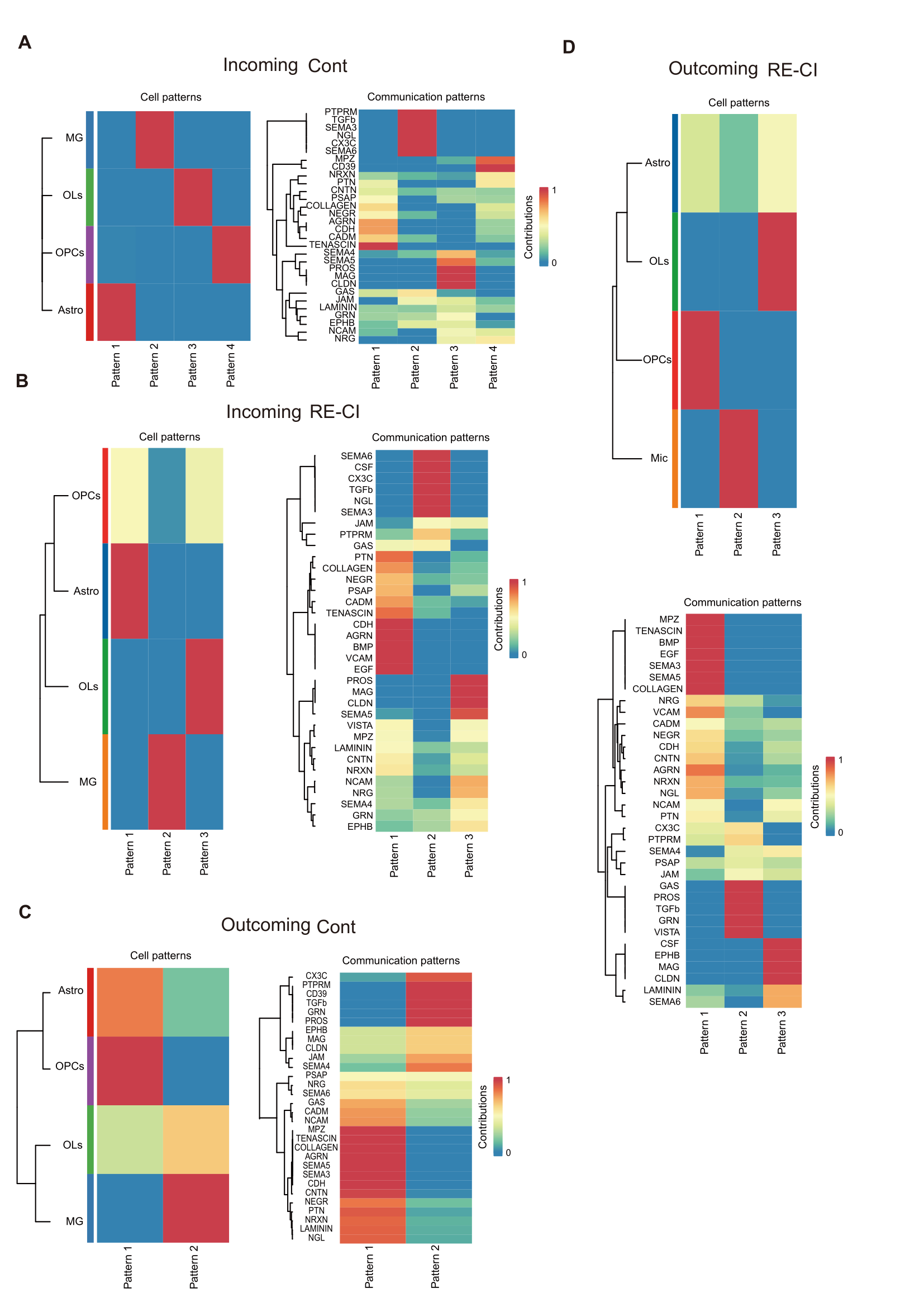

Supplement: Supplementary file 6 — High resolution image (TIF 14.7 MB) [file 12035_2025_5206_MOESM3_ESM.tiff]

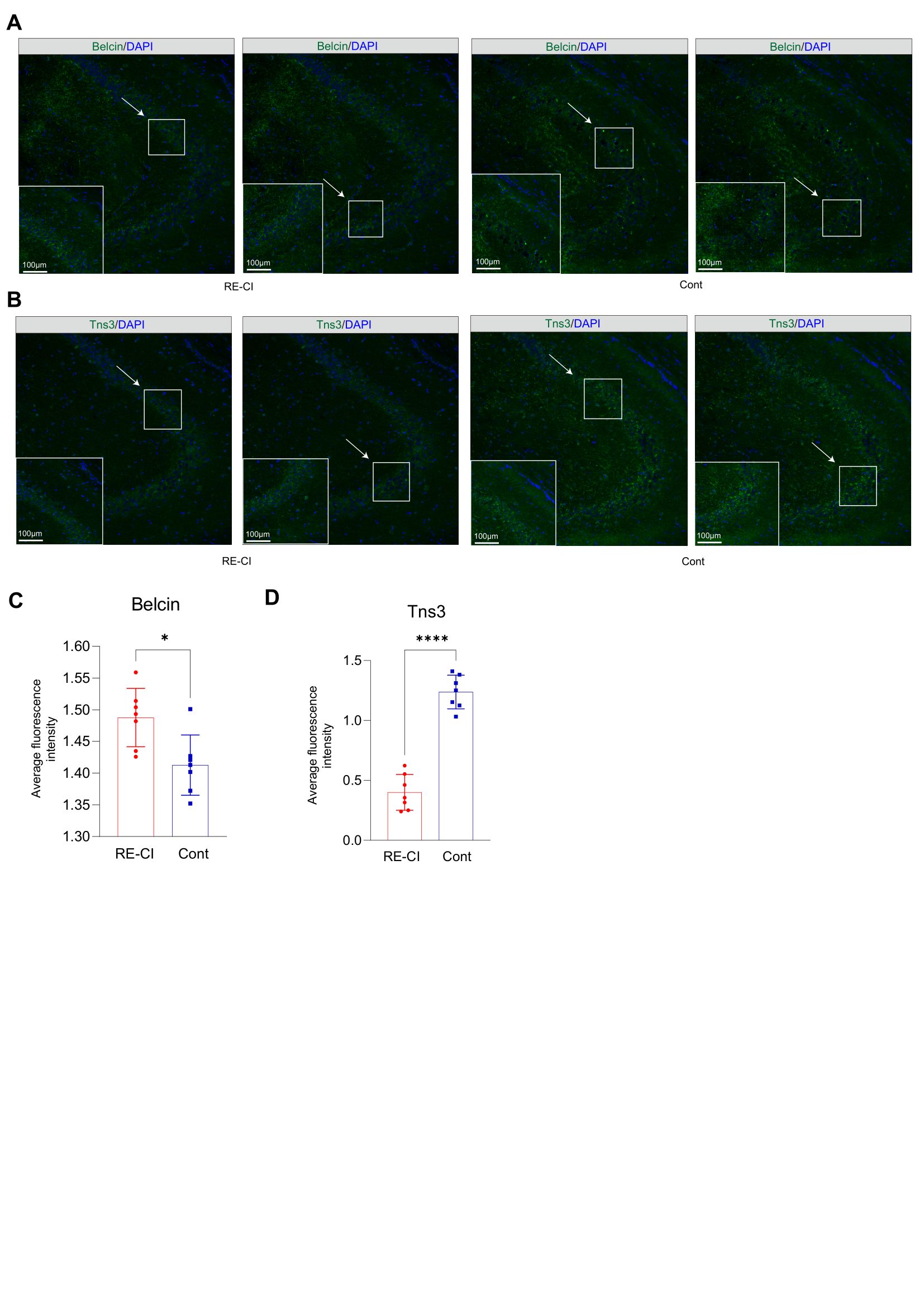

Supplement: Supplementary file 7 — Supplementary figure 12 [file 12035_2025_5206_Fig12_ESM.png]

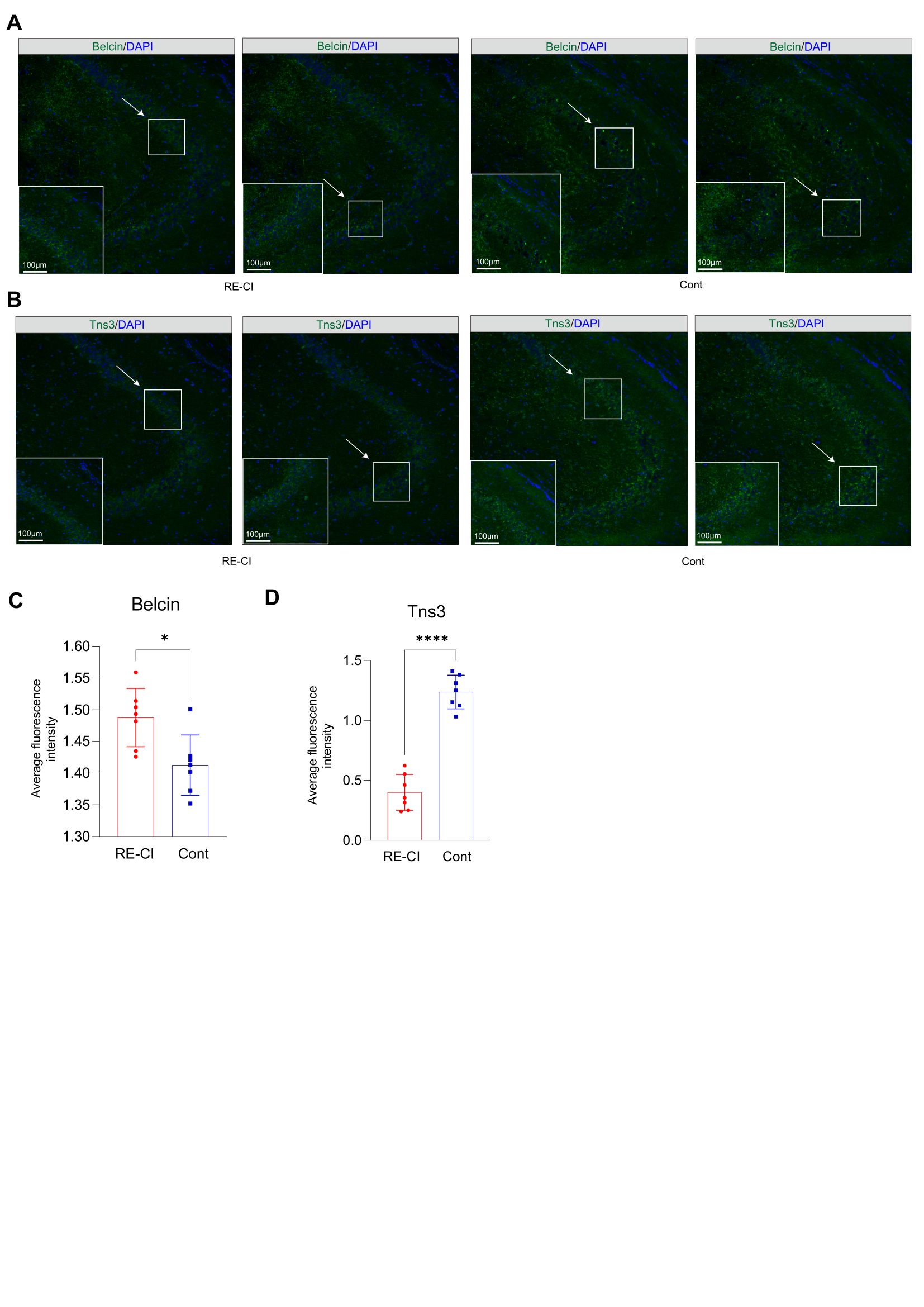

Supplement: Supplementary file 8 — High resolution image (TIF 14.7 MB) [file 12035_2025_5206_MOESM4_ESM.tiff]

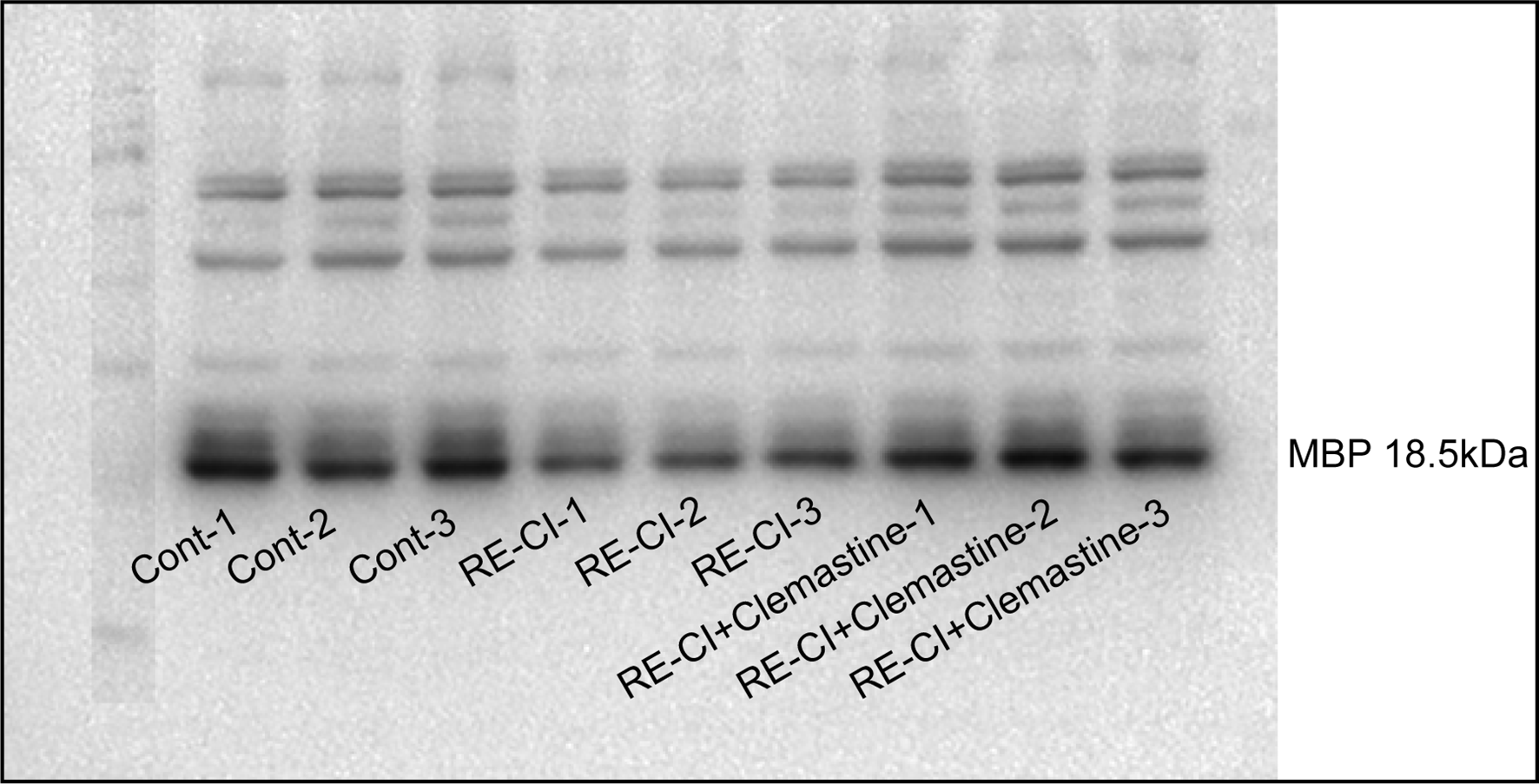

Supplement: Supplementary file 9 — Supplementary figure 13 [file 12035_2025_5206_Fig13_ESM.png]

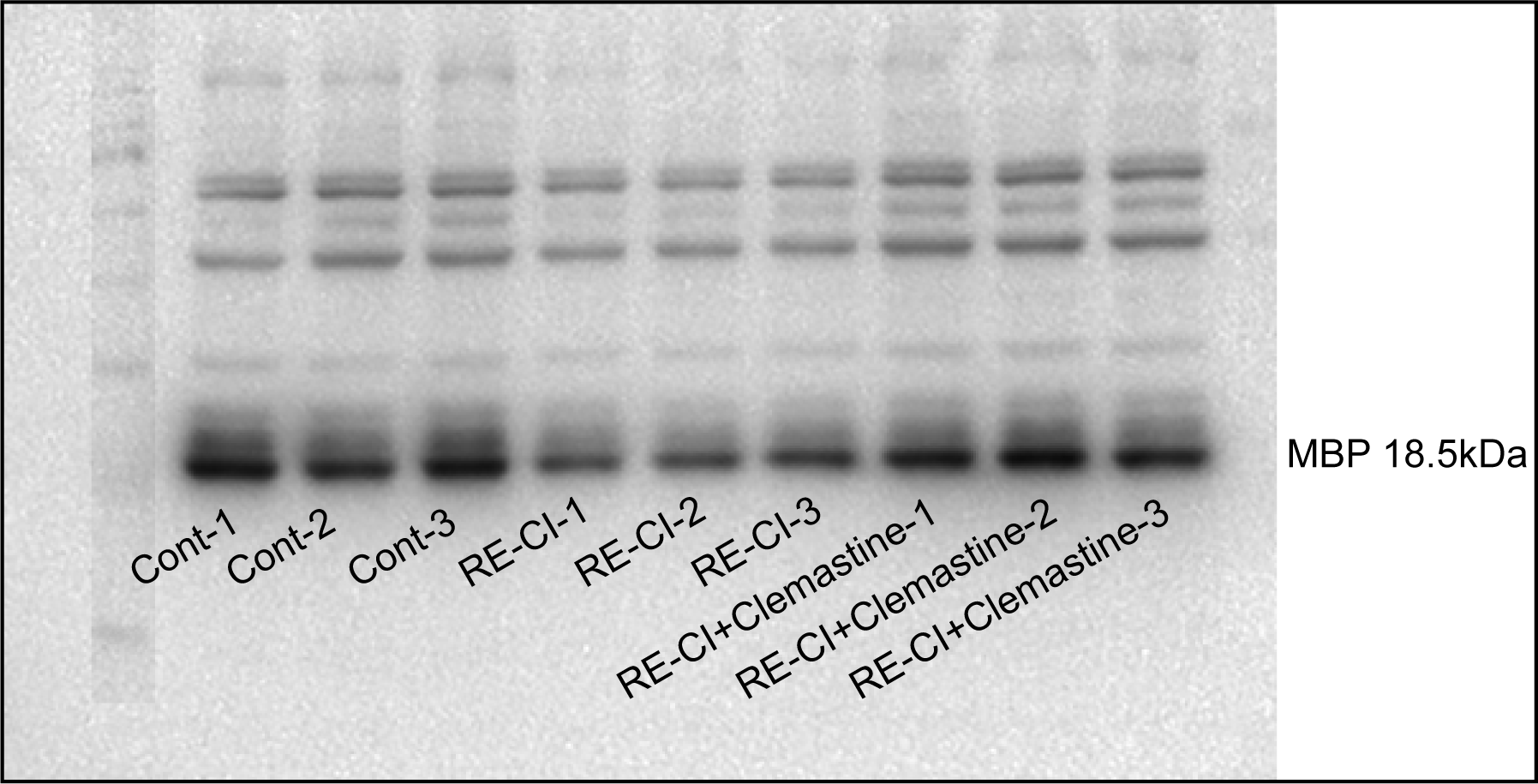

Supplement: Supplementary file 10 — High resolution image (TIF 8.02 MB) [file 12035_2025_5206_MOESM5_ESM.tif]

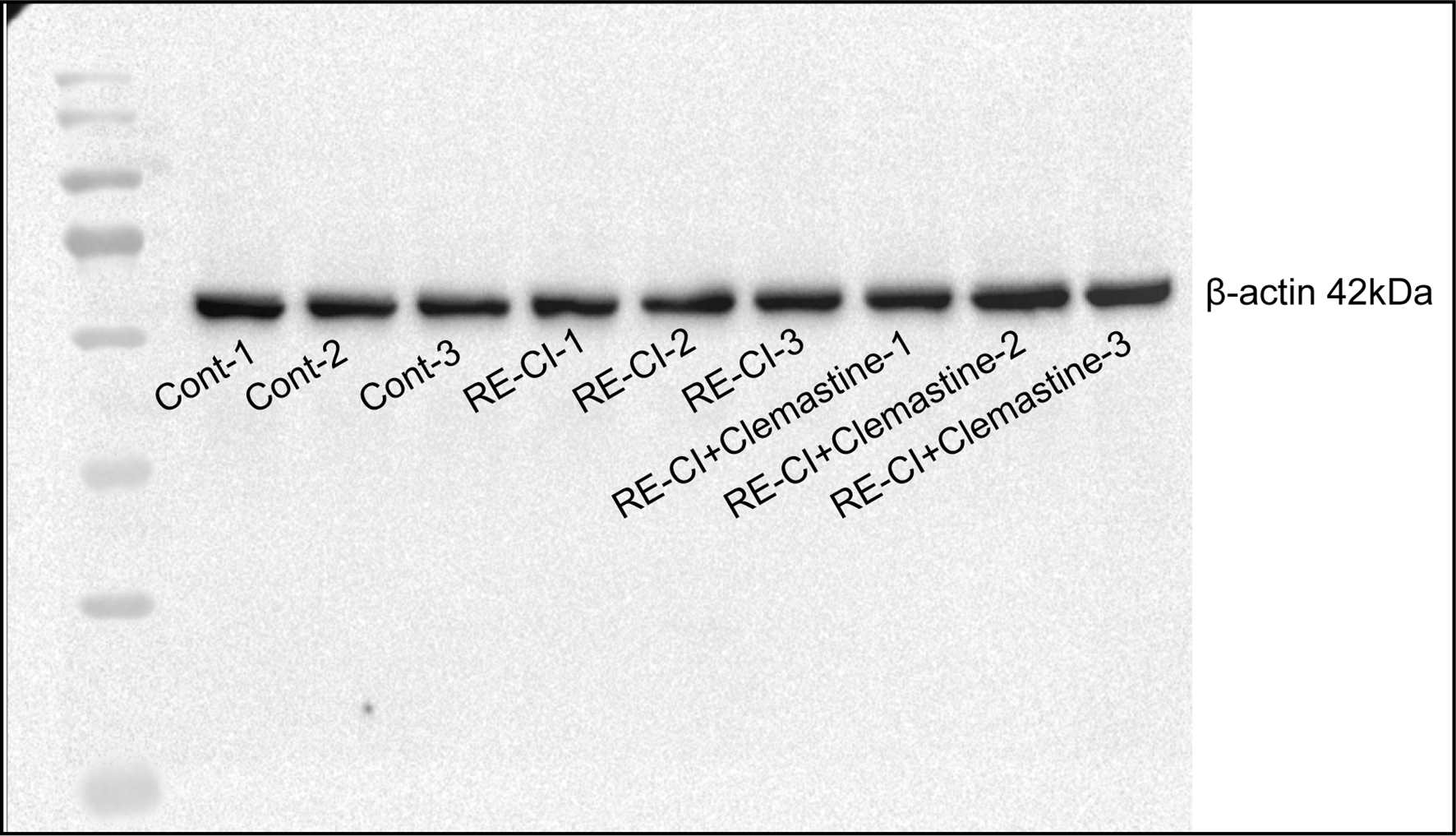

Supplement: Supplementary file 11 — Supplementary figure 14 [file 12035_2025_5206_Fig14_ESM.png]

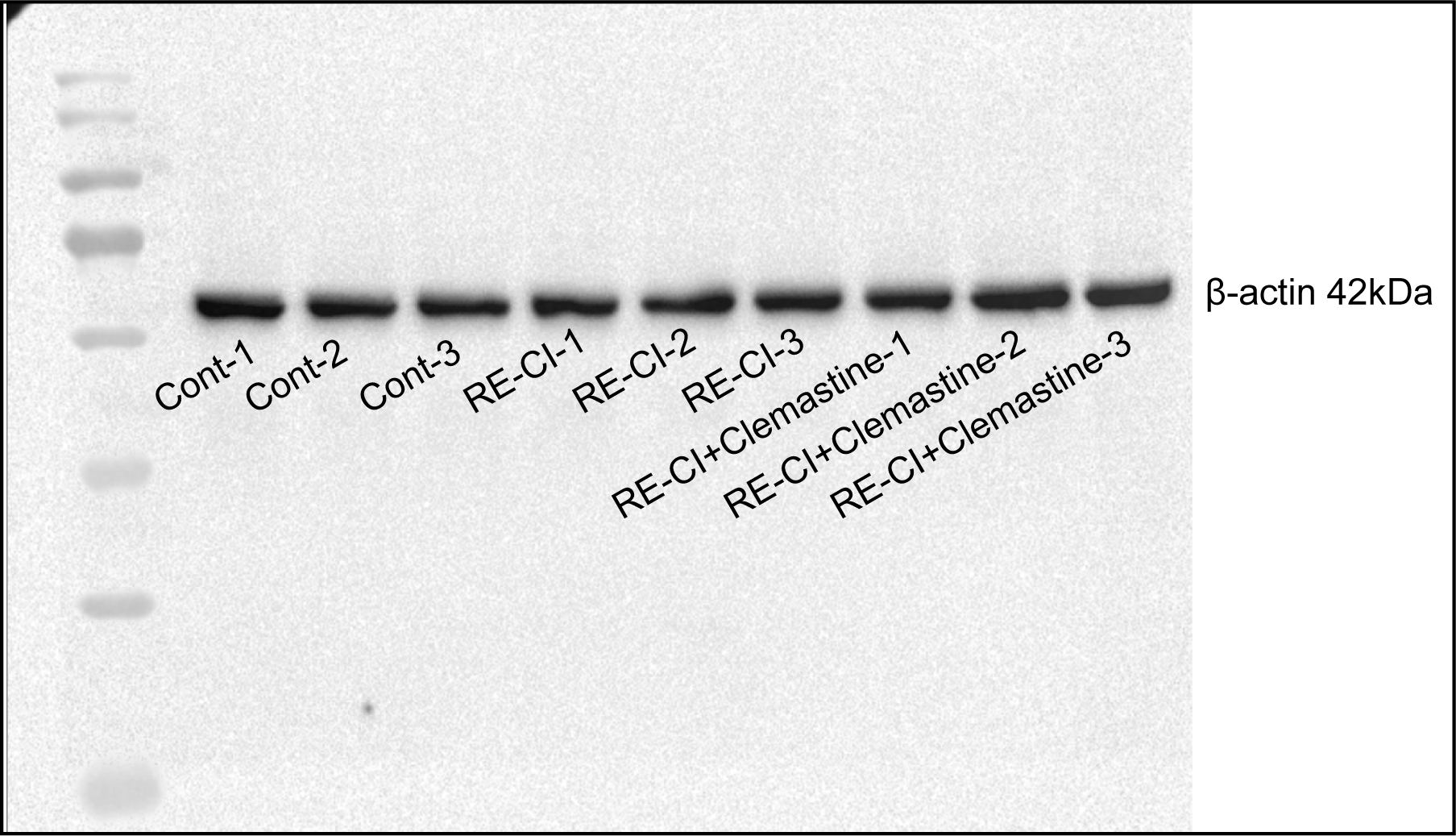

Supplement: Supplementary file 12 — High resolution image (TIF 9.77 MB) [file 12035_2025_5206_MOESM6_ESM.tif]
